# Aligning Environmental Sustainability, Health Outcomes, and Affordability in Diet Quality: A Systematic Review

**DOI:** 10.1016/j.advnut.2023.07.007

**Published:** 2023-07-31

**Authors:** Clarissa L. Leydon, Ursula M. Leonard, Sinéad N. McCarthy, Janas M. Harrington

**Affiliations:** 1 Centre for Health and Diet Research, School of Public Health, University College Cork, Cork, Ireland; 2 Department of Agrifood Business and Spatial Analysis, Teagasc Food Research Centre, Ashtown, Dublin, Ireland; 3 Cork Centre for Vitamin D and Nutrition Research, School of Food and Nutritional Sciences, University College Cork, Cork, Ireland

**Keywords:** diet quality, sustainable diets, food systems, health outcomes, affordability, public health

## Abstract

Improving diet quality while simultaneously maintaining planetary health is of critical interest globally. Despite the shared motivation, advancement remains slow, and the research community continues to operate in silos, focusing on certain pairings (diet–climate), or with a discipline-specific lens of a sustainable diet, rather than examining their totality. This review aimed to summarize the literature on adherence to *a priori* defined dietary patterns in consideration of diet quality, metabolic risk factors for noncommunicable diseases (NCDs), environmental impacts, and affordability. A methodology using PRISMA guidelines was followed, and searches were performed in 7 databases as of October 2022. The Appraisal tool for Cross-Sectional Studies (AXIS) and the National Institutes of Health (NIH) quality assessment tool for observational cohort studies were employed for quality appraisal. The evidence was narratively synthesized according to the characteristics of the diet quality metrics. The review includes 24 studies published between 2017–2023. Thirteen distinct diet quality scores were identified, with those measuring adherence to national dietary guidelines the most reported. Thirteen distinct environmental impact indicators were identified, with greenhouse gas emissions (*n*=23) reported most. All studies reported on body mass index, and 7 studies assessed the cost of adherence. Our results are consistent with previous findings that healthier diets can reduce environmental impacts; however, incongruities between population and planetary health can occur. Hence, the “sustainability” of dietary patterns is dependent on the choice of indicators selected. Further, healthy, lower impact diets can increase financial cost, but may also provide a protective role against the risk of obesity. Given the Global Syndemic, strategies to reduce obesity prevalence should emphasize the win–win opportunities for population and planetary health through dietary change. Research should identify diets that address multiple environmental concerns to curtail burdens potentially transferring, and harmonize this with sociocultural and equity dimensions.

This review was registered at PROSPERO as CRD42021238055.

**Statement of Significance**

This systematic literature review provides up-to-date evidence on adherence to *a priori* defined dietary patterns and the associated environmental impacts. It adds to the body of work in this area and confirms that improving diet quality can reduce diet-related environmental pressures, although not inherently. It also presents population metabolic risk factors for noncommunicable diseases and dietary costs based on adherence.

## Introduction

Food systems exist at an intersection for many overarching global goals and are the nexus that links food security, nutrition, human health, planetary health, and social justice [1]. Food systems are in a precarious position due to climate change, depletion of natural resources, and meeting the demand for safe and nutritious food for an increasing global population [2]. Substantive evidence exists on the contribution of food production to anthropogenic greenhouse gas emissions (GHGE) [[3], [4], [5], [6], [7]], the use of freshwater, land, and fossil fuels [[8], [9], [10], [11], [12], [13]], soil acidification and eutrophication of water bodies [14,15]. In addition, food production contributes to water and air pollution [6,[16], [17], [18]], accelerates biodiversity loss [19,20], and leads to deforestation [21,22], and the overexploitation of fish stocks [23].

Concurrently, food systems affect population health through their impact on food environments and subsequently diet quality [24]. Suboptimal diets are the greatest global challenge of our time and a significant risk factor for the burden of disease [25,26]. Unprecedented levels of diet-related diseases are occurring, with 2 billon adults living with either overweight or obesity [27]. The latest Global Burden of Disease assessment estimated that 8 million deaths were attributable to dietary risk factors [28]. Hence the current food system model is a driver of obesity, undernutrition, and climate change, termed the “Global Syndemic” [29].

Given the current food system’s role in the dual burden of noncommunicable diseases (NCDs) and climate change, the importance of transitioning to an ecosystem-protecting model that prioritizes the provision of healthy sustainable food is undisputable [27,30]. Studies have outlined the adverse associations between diets that are rich in animal-sourced foods and human and environmental health. As such, adoption of sustainable diets that are predominantly plant-rich has been proposed as a solution to improve population and planetary health, and ensure social equity and financial viability for all food system actors [4,8,18,27,31,32]. In addition, population level dietary changes will help achieve many targets of the Sustainable Development Goals [1,4,33,34].

Multiple metrics that measure the healthiness of dietary patterns have been created. Two approaches to defining dietary patterns can be distinguished: *a posteriori* and *a priori*. The *a posteriori* approach derives dietary patterns through statistical methods and therefore, specific to the population they are calculated from, are data-driven rather than recommendation-driven [35]. An *a priori* dietary pattern is based on predefined algorithms to quantify food and nutrient intake based on existing knowledge about the relationships between food, nutrients, and disease [35].

Some *a priori* dietary patterns, well established for positive health outcomes, have been examined alongside environmental impacts. Research has shown that high accordance to the Dietary Approaches To Stop Hypertension (DASH) diet had lower GHGE compared to least-accordant diets in the United Kingdom [36]. Conversely, the DASH diet was not linearly associated with environmental sustainability in Italy [37].

On the economic dimension of a sustainable diet, cost as a proxy for affordability, has been evaluated with diet quality scores. Greater accordance with the DASH diet was found to increase dietary costs [36]. Moreover, healthy diets remain unaffordable for many [38]. Therefore, the aim of this paper was to systematically review the literature on adherence to *a priori* defined dietary patterns, based on quantifiable dimensions of a sustainable diet, namely diet quality, metabolic risk factors for NCDs, environmental impacts, and affordability, where reported.

## Methods

### Design

This review follows the PRISMA standardized reporting guidelines [39]. The review protocol was prospectively registered with PROSPERO on 23 March, 2021 (ID CRD42021238055).

### Search strategy

Initial key word search terms were selected based on author consensus through identification of terms used in reviews of a similar nature [[40], [41], [42], [43]]. A combination of key word searches and Medical Subject Headings (MeSH) (or equivalent) were used across 3 concepts; *1*) diet quality indices or scores, *2*) environmental sustainability, and *3*) health outcomes, along with Boolean logic modified to each database. The search strategy was piloted before final searches were run. The full search strategy is available in Supplementary Table S1.

Seven databases were searched on 1 April, 2021: PubMed (US National Library of Medicine), Web of Science (Clarivate Analytics), Scopus (Elsevier), Embase (Elsevier), Greenfile (EBSCOhost), CINAHL Plus (EBSCOhost), and Cochrane Library. Database searches were limited to the English language and a publication limit from the year 2000. A final search was run just before data synthesis to collect published studies as of 20 October, 2022. All searches were conducted by the primary reviewer (CL). All records were imported to reference manager software Zotero (version 6) [44] and de-duplicated. The remaining records were imported to Rayyan, a web-based tool for screening research articles in collaborative and blinded systematic reviews [45].

### Study inclusion and exclusion criteria

Each article was assessed for eligibility based on predefined criteria outlined in Table 1.

**TABLE 1:** Eligibility criteria

Inclusion	Exclusion
Free-living, healthy adults ≥18 y old	Individuals with a pre-existing medical condition (eg, type 1 diabetes, cancer) excluding diet-related diseases or individual <18 y old
*A priori* (investigator-defined dietary patterns) food-based diet quality metric	Data-driven dietary metrics; metrics calculated based solely on nutrients, eg, PANDiet score; metrics that, in addition to diet, have more than 2 components on lifestyle behaviors such as physical activity; metrics that include components beyond diet and health, eg, Sustainability Diet Index
Adherence score for the population or subgroup to the diet quality metric	Description of adherence or percentage of the metric components achieved
Quantitative assessment of the dietary environmental impact(s)	No quantitative assessment
Anthropometric marker(s) in the appropriate unit of measure (eg, BMI – kg/m^2^) OR cardiometabolic risk biomarker(s) (eg, blood pressure – mmHg)	No quantitative anthropometric or cardiometabolic risk factor
Study design, abstract, and full text available	Abstract, conference proceedings, gray literature, books, and review articles

Abbreviations: BMI, body mass index; PANDiet, Probability of Adequate Nutrient Intake.

### Study selection

Titles and abstracts of studies were independently screened by 2 reviewers (CL and UL). Following the retrieval of full texts, 2 reviewers (CL and UL) independently screened them for inclusion, with adjudication by a third reviewer (JH), where necessary. Neither reviewer was blinded to the journal titles or the study authors. After selecting eligible articles, one reviewer (CL) carried out backward reference searching to identify additional studies.

### Data extraction

A tailored data extraction form was developed and piloted for this study. Data were extracted by one reviewer (CL) and cross-checked by a second (UL). Data were extracted for the outcomes of interest (diet quality, environmental impact, metabolic risk factor(s), and financial cost), in addition to other relevant information (see Supplementary File 2).

### Quality assessment

Two methodological quality assessment tools were utilized in this review. The first was the Appraisal tool for Cross-Sectional Studies (AXIS) [46] which comprised of a 20-item checklist that requires a “yes,” “no,” or “don’t know” response. The second was the NIH quality assessment for observational cohort studies [47]. This tool is comprised of a 14-item checklist that requires a “yes,” “no,” or “cannot determine/not applicable” response. An overall subjective rating of quality (low, fair, or good) was assigned to each study. The primary reviewer (CL) conducted the assessment with cross-checks completed by a second (UL).

### Data synthesis

Given the heterogeneity of the studies, a formal meta-analysis was not possible. A narrative synthesis, based on aggregates of the diet quality metrics’ characteristics, was deemed appropriate to answer the research question. Interactions between environmental impacts, health outcomes, diet quality, and cost were evaluated and presented quantitatively in summary tables, where appropriate. The direction of impact (higher or lower) for environmental indicators, metabolic risk factors, and monetary cost according to diet quality was described to answer the overarching question: *How does differentiation in adherence to a priori dietary patterns align with environmental, health, and affordability outcomes?*

## Results

### Overview of the search and selection process

**FIGURE 1:**
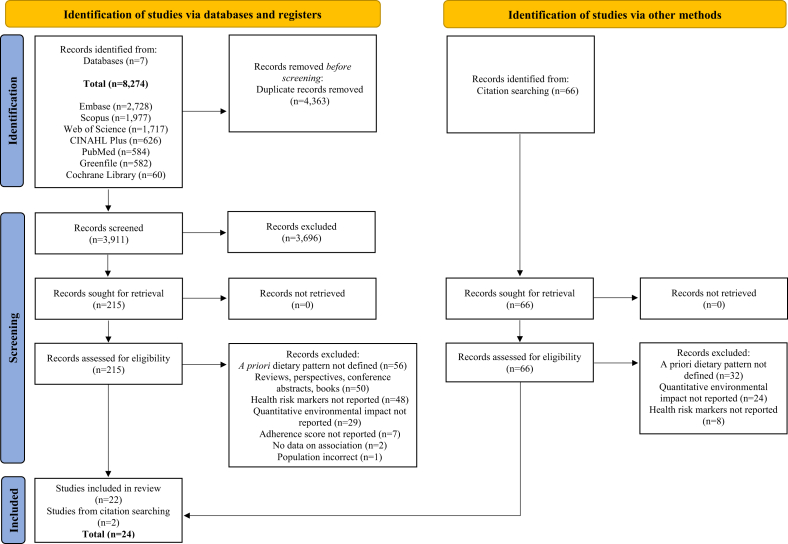
PRISMA 2020 flow diagram of the identification, screening, and selection process for included articles [48].

A total of 8274 articles were retrieved, with 4363 duplicates removed. The remaining articles were screened based on title and abstract. Subsequently, 215 articles qualified for full-text review, and 193 articles were excluded for not meeting inclusion–exclusion criteria, see Figure 1. Efforts were made to contact authors of eligible studies without complete results, to no avail. Backward reference searching identified 2 additional studies, bringing the total to 24 articles.

### Study characteristics

In total, 22 studies employed a cross-sectional analysis and 2 a longitudinal analysis, and were published between 2017 and 2023. With the exception of 2 studies from Australia, all other studies were based in Europe (*n*=22). The FFQ (*n*=11) and 24-h dietary recall (*n*=8) were the most frequent dietary assessment methods used (Table 2). Results of the quality assessment are provided in Supplementary File 1. Some studies reported receiving funding from industry [[49], [50], [51]], and 1 declared previous connections to industry [53]. All studies were included in the review, irrespective of the quality assessment decision by authors.

**TABLE 2:** Overview of studies included in the systematic review

Authors, date, country, reference	Study design	Population characteristics	Dietary data	Dietary assessment method	A priori diet quality metric	Environmental impact reported	Health outcome reported	Dietary cost reported
Perraud et al. 2023, France, [58]	Cross-sectional analysis	1125 participants Male 50.1% Age 39.9 ± 13.2	The third French individual and national food consumption (INCA3) survey 2014–2015	3 non-consecutive 24-h dietary recalls	The Literature-Based Adherence Score to the Mediterranean Diet (LAMD) score The Alternative Healthy Eating Index (AHEI-2010) Programme National Nutrition Santé – Guidelines Score 2 (PNNS-GS2)	GHGE Ionizing radiation Ozone depletion Photochemical ozone Particulate matter Acidification Terrestrial eutrophication Freshwater eutrophication Marine eutrophication Freshwater ecotoxicity Land use Water use Energy use Metal and mineral use	BMI	No

Kesse-Guyot et al., 2022, France, [54]	Cross-sectional analysis	29,326 participants Male 50% Age 54.5 ± 14.1	NutriNet-Santé Cohort 2014	Semi-quantitative organic FFQ	Diet Quality Index (cDQI) Programme National Nutrition Santé – Guidelines Score 2 (PNNS-GS2)	GHGE Cumulative energy demand Land occupation pReCiPe	BMI	Yes

Marty et al., 2022, France, [55]	Cross-sectional analysis	938 participants Female 78.5% Age 39 ± 12	Pre-registered online survey 2020	FFQ	Programme National Nutrition Santé – Guidelines Score 2 (PNNS-GS2) EAT-Lancet Diet Index (ELD-I)	GHGE	BMI	No

Marty et al., 2022, France, [70]	Longitudinal analysis	524 participants Female 79.6% Age 39.5 ± 12.0	Pre-registered online survey 2020–2021	FFQ	Programme National Nutrition Santé – Guidelines Score 2 (PNNS-GS2)	GHGE	BMI	No

Ridoutt et al., 2022, Australia [49]	Cross-sectional analysis	9341 participants Male 45.8% Age 45.5	Australian Health Survey (AHS) 2011–2013	1 24-h dietary recall	Dietary Guideline Index (DGI)	GHGE Water-scarcity impact Cropland-scarcity Pesticide-toxicity footprint	BMI	No

Frehner et al., 2021, Switzerland, [73]	Cross-sectional analysis	2057 participants Male 45.4% Age 46.1 ± 15.4	Swiss National Nutrition Survey (menuCH) 2014–2015	2 non-consecutive 24-h dietary recalls	Alternate Healthy Eating Index (AHEI)	GHGE Cropland occupation Grassland occupation	BMI	Yes

Heerschop et al., 2021, The Netherlands, [65]	Cross-sectional analysis	2078 participants Male 50.2% Age 51 (31–70)	Dutch National Food Consumption Survey (DNFCS) 2012–2016	2 non-consecutive 24-h dietary recalls	Dutch Healthy Diet Index 2015 (DHD15-Index)	GHGE Blue water use	BMI	No

Kesse-Guyot et al., 2021, France, [62]	Cross-sectional analysis	29,210 participants Female 75% Age 53.5 ± 14.0	NutriNet-Santé Cohort 2014	Semi-quantitative organic FFQ	EAT-Lancet Diet Index (ELD-I)	GHGE Cumulative energy demand Land occupation pReCiPe	BMI	Yes

Laine et al., 2021, 10 European countries^1^ [74]	Cross-sectional analysis	443,991 Female 71% Age 52 ± 10	European Prospective Investigation into Cancer and Nutrition (EPIC) 1991–2000	Extensive quantitative dietary questionnaires Semi-quantitative organic FFQ Short non-quantitative FFQ 7-d record 14-d record of hot meals	EAT-Lancet Diet (ELD) Score	GHGE Land use	BMI	No

Ridoutt et al., 2021, Australia, [51]	Cross-sectional analysis	9341 participants Male 45.8% Age 45.5	Australian Health Survey (AHS) 2011–2013	1 24-h dietary recall	Dietary Guideline Index (DGI)	GHGE	BMI	No

Telleria Aramburu et al., 2021, Spain, [56]	Cross-sectional analysis	26,165 participants Male 40.5% Age 20.9 ± 2.1	EHU12/24 cohort 2014-2017	Short FFQ	Healthy Eating Index-2010 (HEI-2010) Mediterranean Diet Score (MDS)	GHGE	BMI Body fat %	No

Hobbs et al., 2020, United Kingdom, [53]	Cross-sectional analysis	1655 participants Male 40.9% Age 42.7 ± 12.5	UK National Diet and Nutrition Survey (NDNS) years 1–4 2008/2009–2011/2012	3- or 4-d food diary	Alternative Healthy Eating Index (AHEI)	GHGE Eutrophication potential Acidification potential	BMI Waist and hip circumference Blood pressure Serum cholesterol Glucose	Yes

Kesse-Guyot et al., 2020, France, [57]	Cross-sectional analysis	28,340 participants Male 24.4% Age 49.9 ± 15.9	NutriNet-Santé Cohort 2014	Semi-quantitative organic FFQ	Programme National Nutrition Santé – Guidelines Score 1 (PNNS-GS1) Programme National Nutrition Santé – Guidelines Score 2 (PNNS-GS2)	GHGE Cumulative energy demand Land occupation pReCiPe	BMI	Yes

Van Bussel et al., 2020, The Netherlands, [66]	Cross-sectional analysis	2106 participants Male 50.1% Age 19–30 y 21.5% 31–50 y 44.7% 51–69 y 39.9%	Dutch National Food Consumption Survey (DNFCS) 2007–2010	2 non-consecutive 24-h dietary recalls	Dutch Healthy Diet Index 2015 (DHD15-Index)	GHGE	BMI	No

Baudry et al., 2019, France, [71]	Cross-sectional analysis	29,210 participants Female 75% Age 53.5 ± 14.0	NutriNet-Santé Cohort 2014	Semi-quantitative organic FFQ	Programme National Nutrition Santé – Guidelines Score 1 (PNNS-GS1)	GHGE Cumulative energy demand Land occupation	BMI	Yes

Biesbroek et al., 2019, The Netherlands, [67]	Longitudinal analysis	8932 participants Female 79.4% Age (baseline) Males 44 Female 51	European Prospective Investigation into Cancer and Nutrition – Netherlands (EPIC-NL) Baseline: 1993–1997 Follow-up: 2015	FFQ	Dutch Healthy Diet Index 2015 (DHD15-Index)	GHGE	BMI	No

Mertens et al., 2019, The Netherlands, [52]	Cross-sectional analysis	1169 participants Male 51.9% Age 53.2 ± 11.5	Nutrition Questionnaires plus (NQplus) 2011–2013	2 non-consecutive 24-h dietary recalls Semi-quantitative FFQ	Dutch Healthy Diet Index 2015 (DHD15-Index)	GHGE Fossil energy use Land use pReCiPe	BMI	No

Van Bussel et al., 2019, The Netherlands, [50]	Cross-sectional analysis	1380 participants Male 54% Age 53 ± 12	Nutrition Questionnaires plus (NQplus) 2011–2013	2 non-consecutive 24-h dietary recalls	Dutch Healthy Diet Index 2015 (DHD15-Index)	GHGE Fossil energy use Land use pReCiPe	BMI	No

Vellinga et al., 2019, The Netherlands, [68]	Cross-sectional analysis	2078 participants Males 50.2% Age 48 ± 21 (male), 48 ± 21 (female)	Dutch National Food Consumption Survey (DNFCS) 2012–2016	2 non-consecutive 24-h dietary recalls	Dutch Healthy Diet Index 2015 (DHD15-Index)	GHGE Blue water use	BMI	No

Biesbroek et al., 2018, The Netherlands, [69]	Cross-sectional analysis	36,209 participants Female 73.7% Age 48.6 ± 0.1	European Prospective Investigation into Cancer and Nutrition – Netherlands (EPIC-NL) 1993-1997	FFQ	Dutch Healthy Diet Index 2015 (DHD15-Index)	GHGE	BMI	No

Murakami & Livingstone, 2018, United Kingdom, [59]	Cross-sectional analysis	3502 participants Male 49.2% Age 47.6 ± 17.7	National Diet and Nutrition Survey (NDNS) 2008/2009–2013/2014	4 consecutive food diaries	Healthy Diet Indicator (HDI) Mediterranean Diet score (MDS) Dietary Approaches To Stop Hypertension (DASH)	GHGE	BMI	No

Seconda et al., 2018, France, [72]	Cross-sectional analysis	34,193 participants Female 75.5% Age 48.1 ± 16.3	NutriNet-Santé Cohort 2014	Semi-quantitative organic FFQ	Programme National Nutrition Santé – Guidelines Score 1 (PNNS-GS1)	GHGE Primary energy consumption Land occupation	BMI	Yes

Biesbroek et al., 2017, The Netherlands, [60]	Cross-sectional analysis	35,031 participants Male 26.2% Age^2^	European Prospective Investigation into Cancer and Nutrition – Netherlands (EPIC-NL) 1993-1997	FFQ	Healthy Diet Indicator (HDI) Dietary Approaches To Stop Hypertension (DASH) Dutch Healthy Diet Index 2015 (DHD15-Index)	GHGE Land use	BMI	No

Rosi et al., 2017, Italy, [64]	Cross-sectional analysis	153 participants Females 58.2% Age OMNI 37 ± 9 OVO 39 ± 9 VEG 37 ± 10	Observational multi-center study across 4 geographically distant cities in Italy	consecutive 7-d weighted food record	Italian Mediterranean Diet Index (IMDI)	GHGE Water footprint Ecological footprint	BMI	No

Abbreviations: BMI, body mass index; GHGE, greenhouse gas emissions; OMNI, omnivores; OVO, ovo-lacto-vegetarians; pReCiPe, Partial ReCiPe; VEG, vegans.

Age values presented as mean; mean ± standard deviation (SD); median & interquartile range (IQR); percentage (%)

1 Countries include: Denmark, France, Germany, Greece, Italy, The Netherlands, Norway, Spain, Sweden, and the United Kingdom.

2 Age by tertiles of HDI, Males T1 43.5 ± 11.0; T2 43.1 ± 10.9; T3 42.0 ± 11.0, Females T1 51.2 ± 11.6; T2 50.7 ± 11.4; T3 50.5 ± 11. 8; tertiles of DASH, Males T1 40.8 ± 11.1; T2 43.1 ± 10.8; T3 45.0 ± 10.5, Females T1 47.1 ± 12.3; T2 51.5 ± 11.1; T3 54.4 ± 9.8; tertiles of DHD15-index, Males T1 40.6 ± 11.1; T2 43.0 ± 11.0; T3 44.7 ± 10.5, Females T1 48.5 ± 11.8; T2 51.4 ± 11.3; T3 52.4 ± 11.4.

### Diet quality metrics

**FIGURE 2:**
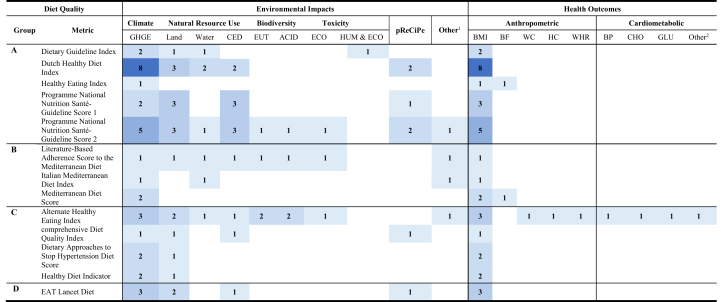
Heat map of diet quality metrics and the various interactions across environmental impacts and health outcomes. Abbreviations: ACID, acidification; BF, body fat; BP, blood pressure; CED, cumulative energy demand; CHO, cholesterol (total cholesterol, high-density lipoprotein [HDL], low-density lipoprotein [LDL]), ECO, freshwater ecotoxicity; EUT, eutrophication; GHGE, greenhouse gas emissions; GLU, glucose (glycated hemoglobin [HbA1c], glucose); HC, hip circumference; HUM & ECO, human toxicity and freshwater ecotoxicity; pReCiPe, partial ReCiPe; WC, waist circumference; WHR, waist-to-hip ratio. Group A: Based on adherence to international nutrition guidelines or national dietary guidelines; Group B: Based on region specific health-improving diets; Group C: Based on dietary patterns to lower risk of chronic disease; Group D: Based on diets which promote population and planetary health. ^1^includes indicators such as ozone depletion, photochemical ozone formation, particulate matter, metals and minerals use, ecological footprint. ^2^includes markers such as triglycerides, C-reactive protein, pulse pressure.

A single diet quality metric was used by most studies, with some using 2 [[54], [55], [56], [57]] or 3 metrics [[58], [59], [60]]. There were 13 distinct diet quality metrics, with modified versions also adopted. Of these, the Dutch Healthy Diet Index 2015 (DHD15-Index) was the most commonly used (*n*=8), followed by the Programme National Nutrition Santé Guidelines Score (PNNS-GS) (*n*=7). Due to variability, the diet quality metrics were grouped as follows: food-based dietary guidelines (FBDG) (A), region-specific healthy diets (B), diets to lower risk of chronic disease (C), and diets promoting population and planetary health (D) (Figure 2).

A description of each diet quality metric reported is provided in Supplementary File 1.

### Environmental impacts

The environmental impact indicator reported by most studies was GHGE (*n*=23), followed by land use or occupation (*n*=11). Land occupation (LO) corresponds to the area involved in the production processes, without considering the duration of the land use, expressed in m^2^. Land use is a measure of the surface of land required over a certain period of time. It is denoted by area of occupation (m^2^) multiplied by the time of occupation (eg, year) [61]. One study reported land use in loss of soil organic matter content, in kilograms of carbon deficit [58], and another reported on cropland-scarcity [49]. A partial ReCiPe (pReCiPe) score was calculated by 5 studies [50,52,54,57,62], which is a synthetic estimate of overall environmental impact based on GHGE, cumulative energy demand (CED), and LO. The pReCiPe enables the consideration of potential trade-offs between indicators, with a high score indicating a greater environmental impact [63]. Two studies assessed toxic impacts of pesticides using the USEtox model. One study on freshwater ecotoxicity [58], and the other aggregated human (carcinogenic and non-carcinogenic) and ecotoxicological impacts [49]. One study reported on the ecological footprint (EF), which is the amount of biologically productive land and sea needed [64]. Only one study reported on a range of environmental impacts (14 indicators in total) [58].

### Health outcomes

All studies reported BMI, both measured (*n*=14) and self-reported (*n*=10) data. One study reported Body Fat (BF) status [56], and another waist circumference, hip circumference, and waist-to-hip ratio [53]. For cardiometabolic risk factors, one study reported blood pressure, cholesterol, glucose, and other markers [53].

The evidence base for all possible combinations of diet quality metrics, environmental impact indicators, and metabolic risk factors is depicted by the number of metrics in Figure 2. The greatest interaction among studies was between the DHD15-Index, GHGE, and BMI.

### Diet quality metric—Group A

#### Dietary Guideline Index

Two Australian studies [49,51] measured adherence to the 2013 dietary guidelines using the Dietary Guideline Index (DGI). Both studies by Ridoutt et al. [49,51], identified subgroups of adults’ diets from the population. The first subgroup is characterized by a “Higher diet Quality and Lower environmental Impact” (HQLI), based on 4 environmental impact indicators [49], and the second, a “Higher diet Quality score and Lower Emissions” (HQLE) [51]. These subgroups had greater adherence to the guidelines; a 39% (HQLI) and 38% (HQLE) higher score compared with the average Australian diet (59.2 (HQLI) and 58.7 (HQLE) versus 42.6 out of 100). The HQLI subgroup had lower environmental impacts compared with the average population for the following footprints: water scarcity (-24%), cropland scarcity (-29%), pesticide toxicity (-34%), and climate (GHGE) (-53%). There were lower GHGE (-43%) observed for the HQLE subgroup. The HQLE subgroup were more likely to be in the normal weight range and less likely to be obese than the overall population. However, no significant difference in BMI was observed for the HQLI subgroup compared with the total population.

#### Dutch Healthy Diet index

Eight studies measured compliance with the Dutch dietary guidelines using the DHD15-Index, and none reported full adherence (Table 3). Those with greater adherence were more likely to be females [52,60,[65], [6]], engaged in healthy lifestyle behaviors [60,69], and obtained a higher level of education [60,[65], [66], [67],^69^]. Although, van Bussel et al. [66] found that GHGE did not significantly differ between education groups, despite higher adherence in the high education group for both males and females. The BMI of males did not differ between education groups, with the opposite found for females.

Some studies suggested that increasing adherence can lower environmental impacts [52,60,67,68]. Mertens et al. [52] found that environmental impacts were inversely associated with the DHD15-Index for both the FFQ and 24-h recall dietary assessment. Females had higher diet quality scores and lower environmental impacts. Biesbroek et al. [67] reported on the differences in Dutch guideline adherence and dietary GHGE over 20 y. The DHD15-Index scores increased between 1993–1997 and 2015 by 11% in males and 13% in females. Dietary GHGE were 2% and 4% lower in males and females in 2015 compared with 1993–1997. However, males had higher (5%) GHGE in 2015 compared to baseline when expressed per 1000 kcal, whereas females had similar relative dietary GHGE. BMI increased at follow-up for males and females. Biesbroek et al. [60] stratified participants based on tertiles of compliance. Greater adherence to the guidelines (T3) resulted in lower GHGE (males 5%; females 5%) and land use (males 7%; females 9%) compared with lower adherence (T1), after adjusting for age, energy intake (EI), and physical activity level (PAL).

Depending on the dietary pattern characteristics, greater adherence can lead to higher or lower impacts [50,69]. van Bussel et al. [50] used sex-specific tertiles to identify 4 subgroups based on combinations of healthiness (DHD15-Index) and environmental impact (pReCiPe score) of diets; “High on Sustainability and High on Health” (High-S&High-H); “High on Sustainability, Low in Health” (High-S&Low-H); “Low on Sustainability, High on Health” (Low-S&High-H) and “Low on Sustainability, Low on Health” (Low-S&Low-H). GHGE were lower in the High-S groups compared with the Low-S groups. Similar findings were observed for land and fossil energy use. Adherence scores were higher in the High-H subgroup compared with the Low-H-subgroups. The High-S&High-H subgroup had a 45%, 46%, and 30% lower GHGE, land use, and fossil energy use, respectively, when compared with the Low-S&High-H subgroup, although adherence to the Dutch Guidelines was similar in both. The second study by Biesbroek et al. [69] identified 2 dietary patterns, “Plant-Based” (PB) and “Dairy-Based” (DB). Quartile 4 (Q4) of the PB pattern had greater compliance to the guidelines and lower GHGE than the DB Q4 diet (adjusted for sex, age, and EI). Both patterns were healthier compared with the average diet, but only the PB pattern had lower emissions.

Two studies reported that higher diet quality may not result in lower impacts across all environmental indicators [65,68]. Heerschop et al. [65] identified 3 patterns: the “High Fruit and Vegetable” (HF&V), “Low Meat” (LMeat), and “High Dairy and Low Fruit Juices” (HD&LFJ). Those in Q4 of each dietary pattern had the highest level of adherence to the Dutch guidelines (Table 3). Those in Q4 of the HF&V pattern had the highest DHD15-Index scores and higher GHGE (14%), and blue water use (69%) compared with the population average. High adherence to the HD&LFJ pattern resulted in higher scores (13%), GHGE (9%), and lower blue water use (8%) compared with the population average. The LMeat Q4 group was the most sustainable pattern, with 20% lower GHGE and 8% higher blue water use. Vellinga et al. [68] found that greater adherence was inversely correlated with GHGE and positively correlated with blue water use (adjusted for sex, age, and EI).

**TABLE 3:** Dutch Healthy Eating Index and the associated dietary environmental impacts and body mass index

Author	Diet quality		Environmental impact							Health outcome
	Adherence score	Cohort grouping	Functional unit	System soundary^8^	GHGE	Water	Land	Energy	pReCiPe score	BMI, kg/m^2^
Heerschop et al., 2021^1^ [65] Modified (0 – 140)	59.4 ± 18.6	Total	GHGE kg CO_2_eq/2000 kcal Blue water m^3^/2000 kcal	Cradle-to-grave	4.70 (4.02–5.62)	0.13 (0.10–0.19)	—	—	—	25.5 (22.7–29.0)
	42.1 ± 13.1	Q1 HF&V			4.26 (3.70–4.98)	0.09 (0.07–0.11)	—	—	—	25.2 (22.2–29.0)
	75.6 ± 15.3	Q4 HF&V			5.36 (4.54–6.33)	0.22 (0.17–0.28)	—	—	—	25.6 (22.9–29.4)
	51.5 ± 17.3	Q1 LMeat			5.98 (5.20–6.96)	0.13 (0.10–0.19)	—	—	—	27.2 (24.2–30.6)
	69.5 ± 17.8	Q4 LMeat			3.78 (3.35–4.22)	0.14 (0.09–0.20)	—	—	—	24.0 (21.7–27.1)
	52.2 ± 19.0	Q1 HD&LFJ			4.52 (3.78–5.49)	0.18 (0.12–0.24)	—	—	—	25.3 (22.7–28.4)
	67.1 ± 16.9	Q4 HD&LFJ			5.13 (4.42–6.05)	0.12 (0.09–0.17)	—	—	—	25.9 (23.3–29.5)

van Bussel et al., 2020^2^ [66] Modified (0 – 140)	60 (0.34)	Total	GHGE kg CO_2_eq/d	Cradle-to-grave	4.30 (0.05)	—	—	—	—	26.2 (0.1)
	55 (0.46)	Male			4.84 (0.08)	—	—	—	—	26.1 (0.1)
	65 (0.46)	Female			3.77 (0.06)	—	—	—	—	26.2 (0.2)
	53 (1)	Male Low Ed			4.92 (0.10)	—	—	—	—	26 (0)
	55 (1)	Male Med Ed			4.80 (0.13)	—	—	—	—	26 (0)
	59 (1)	Male High Ed			4.80 (0.15)	—	—	—	—	26 (0)
	64 (1)	Female Low Ed			3.75 (0.07)	—	—	—	—	27 ((0)
	65 (1)	Female Med Ed			3.77 (0.10)	—	—	—	—	26 (0)
	69 (1)	Female High Ed			3.79 (0.12)	—	—	—	—	25 (0)

Biesbroek et al., 2019^3^ [67] Modified (0 – 120)	64.8 (95% CI 50, 79.9)	Male Baseline	GHGE kg CO_2_eq/d	Cradle-to-grave	5.92	—	—	—	—	25.62 ± 3.18^7^
	65.2 (95% CI 51, 80)	Female Baseline			4.94	—	—	—	—	24.96 ± 3.72
	71.9 (95% CI 41.9, 97.8)	Male Follow-up			5.82	—	—	—	—	26.17 ± 3.88
	73.6 (95% CI 45.3, 99.8)	Female Follow-up			4.74	—	—	—	—	25.68 ± 4.79

Mertens et al., 2019 [52] Complete (0 – 150)	73.8 ± 15	Total	GHGE kg CO_2_eq/d Land use m^2^×y/d Energy demand MJ/d	Cradle-to-grave	3.64 ± 1.46	—	4.15 ± 1.82	31.10 ± 9.20	0.43 ± 0.16	25.6 ± 3.7^7^
	69.2 ± 14.1	Male			3.94 ± 1.60	—	4.57 ± 1.99	33.36 ± 9.83	0.46 ± 0.18	26.2 ± 3.3
	79.4 ± 14.4	Female			3.32 ± 1.20	—	3.71 ± 1.51	28.66 ± 7.77	0.39 ± 0.14	24.9 ± 3.9

Vellinga et al., 2019 [68] Modified (0 – 140)	51.8 ± 22.4	Male	GHGE kg CO_2_eq/d Blue water m^3^/d	Cradle-to-grave	5.98 ± 2.6	0.16 ± 0.11	—	—	—	26.4 ± 6.6
	64.2 ± 23.6	Female			4.58 ± 2.02	0.15 ± 0.11	—	—	—	26.9 ± 7.8

van Bussel et al., 2019^4^ [50] Modified (0 – 140)	64.4 ± 12.6	Total	GHGE kg CO_2_eq/d Land use m^2^×y/d Energy demand MJ/d	Cradle-to-plate	3.7 ± 1.7	—	4.3 ± 2.3	31.0 ± 9.2	0.44 ± 0.20	26 ± 4.0^7^
	79.8 ± 8.4	High S & High H			2.6 ± 0.7	—	2.9 ± 0.9	25.9 ± 5.9	0.31 ± 0.08	25 ± 4.0
	51.8 ± 7	High S & Low H			2.6 ± 0.8	—	2.9 ± 1.0	24.6 ± 7.0	0.31 ± 0.09	27 ± 5.0
	78.4 ± 7	Low S & High H			4.7 ± 1.4	—	5.4 ± 1.8	36.9 ± 10.0	0.54 ± 0.15	25 ± 3.0
	49.0 ± 7	Low S & Low H			5.5 ± 2.3	—	6.7 ± 3.2	37.2 ± 9.7	0.64 ± 0.26	27 ± 4.0

Biesbroek et al., 2018^5^ [69] Modified (0 – 140)	76.2	Total	GHGE kg CO_2_eq/d	Cradle-to-grave	4.06	—	—	—	—	25.6 ± 0.02^7^
	59.9	Q1 Plant			4.29	—	—	—	—	26.3 ± 0.04
	89.9	Q4 Plant			3.96	—	—	—	—	24.9 ± 0.04
	71.7	Q1 Dairy			3.84	—	—	—	—	25.6 ± 0.04
	77.9	Q4 Dairy			4.43	—	—	—	—	25.6 ± 0.04

Biesbroek et al., 2017^6^ [60] Modified (0 – 140)	67.4 ± 16.3	Male	GHGE kg CO_2_eq/d Land use m^2^×y/d	Cradle-to-grave	4.6 ± 0.1	—	4.4 ± 0.1	—	—	25.6 ± 3.2^7^
	80.0 ± 14.9	Female			3.7 ± 0.1	—	4.4 ± 0.1	—	—	25.0 ± 3.7
	49.8 ± 7.7	Male T1			4.74 ± 0.1	—	4.52 ± 0.1	—	—	26.0 ± 3.8
	66.9 ± 4.1	Male T2			4.64 ± 0.1	—	4.40 ± 0.1	—	—	25.8 ± 3.4
	85.6 ± 9.0	Male T3			4.48 ± 0.1	—	4.20 ± 0.1	—	—	25.4 ± 3.2
	63.6 ± 8.1	Female T1			3.82 ± 0.1	—	3.61 ± 0.1	—	—	25.9 ± 4.4
	80.3 ± 3.7	Female T2			3.76 ± 0.1	—	3.46 ± 0.1	—	—	25.6 ± 4.0
	96.1 ± 7.3	Female T3			3.63 ± 0.1	—	3.27 ± 0.1	—	—	25.0 ± 3.8

Abbreviations: BMI, body mass index; GHGE, greenhouse gas emissions; HD&LFJ: High dairy, low fruit juices dietary pattern; HF&V, high fruits and vegetables dietary pattern; LMeat, low meat (red and processed meat) dietary pattern; pReCiPe, partial ReCiPe; Q, quintile; T, tertile.

Values are presented as mean; mean ± standard deviation (SD); median & interquartile range (IQR); mean & standard error (SE); mean & 95% confidence interval (CI)

1 HF&V: Q1 79 g; Q4 699 g/2000 kcal; LMeat: Q1 134 g; Q4 13 g/2000 kcal; HD&LFJ: dairy - Q1 149 g; Q4 453 g/2000 kcal, fruit juice - Q1 126 g; Q4 0 g/2000 kcal

2 Low ED: low education level (primary school, lower vocational, low or intermediate general education); Med ED: medium education level (intermediate vocational education and higher general education); High ED: High education level (higher vocational education and university)

3 Baseline (1993–1997); follow-up (2015)

4 Subgroups are based on sex-specific tertiles of DHD15-Index and pReCiPe score; High-S & High-H: high on sustainability, high on health; High-S & Low-H: high on sustainability, low in health; Low-S & High-H: low on sustainability, high on health; Low-S & Low-H: low on sustainability, low on health; Health refers to adherence to the Dutch dietary guidelines; sustainability refers to the environmental impact

5 Plant: plant-based dietary pattern (Q1 high consumption of fries, sugar sweetened beverages, alcoholic beverages, red and processed meat [93 g/d]; Q4 high consumption of fruits, vegetables [F&V 439 g/d], soy products, legumes, cake and pies, and fish); Dairy: dairy-based dietary pattern (Q1 high consumption of coffee and tea [914 g/d], sugar sweetened beverages, cereals, soy products; Q4 high consumption of cheese, dairy [523 g/d], nuts, and seeds)

6 Tertiles of DHD15-Index adherence (Males: T1 ≤59.9; T2 60.0–74.2; T3 ≥74.2; Females: T1 ≤73.7; T2 73.8–86.7; T3 ≥86.7)

7 Measured BMI

8 System boundary as defined by study authors

For BMI, 2 studies reported that among participants with higher quality and lower environmental impact diets, lower BMI was observed [60,69]. One study reported this among females only [52]. Lower BMI was found among those following dietary patterns with lower impacts only [65] or those with greater diet quality only [50].

#### Healthy Eating Index

Telleria-Aramburu et al. [56] measured adherence to the 2010 American dietary guidelines using the Healthy Eating Index (HEI) and reported a score of 74.48 out of 100 in the population, with females having lower GHGE than males. Those in the high-GHGE diet group (>5.78 kg CO_2_eq/d) had higher HEI scores (13%) than those with low-GHGE diets (low-GHGE diet; <3.39 kg CO_2_eq/d), even when controlling for sex, socioeconomic status (SES) and BF status. Further, an inverse association between diet quality and BF was found.

#### Programme National Nutrition Santé Guidelines score

Seven studies reported adherence to the French national recommendations of the Programme National Nutrition Santé for 2001 (PNNS-GS1) or 2017 (PNNS-GS2) [54,55,57,58,[70], [71], [72]]. Three studies used the PNNS-GS1 [57,71,72], and 2 reported lower environmental impacts with higher diet quality scores [57,71], see Table 4. The first, by Kesse-Guyot et al. [57], categorized participants into sex-specific weighted quintiles reflecting the level of adherence to the 2001 guidelines. After adjustment for EI, lower environmental pressures were associated with a higher level of dietary guideline adherence, with a decrease of 25% in the pReCiPe score, although energy demand increased with adherence (7%). The second study by Baudry et al. [71] stratified participants according to the proportion of organic food in their diet. Those in Q5 (71% of the diet), had greater adherence to the guidelines and lower impacts, compared to those with no organic food consumption (Q1) (adjusted for age, sex, and daily EI). Environmental impacts reduced as follows: GHGE 37%, land use 23%, and energy use 26%, compared with those in Q1.

**TABLE 4:** Programme National Nutrition Santé Guidelines Score and the associated dietary environmental impacts and body mass index

Author	Diet quality		Environmental impact								Health outcome
	Adherence score	Cohort grouping	Functional unit	System boundary^10^	GHGE	Water	Land	Energy	pReCiPe score	Freshwater ecotoxicity	BMI, kg/m^2^
Perraud et al., 2023^1^ [58] sPNNS-GS2 (-17 to 13.5)	6.3 ± 1.8	Total	GHGE kg CO_2_eq/d Water m^3^/d Land use kg C deficit/d Energy demand MJ/d Freshwater ecotoxicity^9^ CTUe/d	Cradle-to-plate	6.4 ± 3.3	6.7 ± 3.2	314.4 ± 58.4	61.8 ± 21.9	—	151.4 ± 58.4	24.9 ± 4.6^8^
	5.9 ± 1.6	P1			4.6 ± 1.9	6.0 ± 94.9	210.3 ± 7.4	53.7 ± 55.1	—	132.2 ± 0.5	24.1 ± 4.4
	6.1 ± 2.1	P2			5.6 ± 2.6	6.2 ± 156.9	283.5 ± 9	56.5 ± 53	—	140.8 ± 0.4	24.4 ± 4.5
	6.4 ± 1.8	P3			6.6 ± 3.4	7.1 ± 199.2	309.2 ± 11.4	61.9 ± 52.7	—	146.2 ± 0.8	25.0 ± 4.7
	6.0 ± 1.5	P4			7.7 ± 4.5	5.9 ± 251.2	396.7 ± 13.6	58.4 ± 60.5	—	151.2 ± 0.5	24.8 ± 5
	6.5 ± 1.7	P5			6.4 ± 2.9	6.9 ± 175.3	319.6 ± 14.2	67.1 ± 61.7	—	164.2 ± 0.5	25.3 ± 4.4

Kesse-Guyot et al., 2022^2^ [54] sPNNS-GS2 (-17 to 14.25)	5.64 ± 1.97	LAC	GHGE kg CO_2_eq/d Land occupation m^2^/d Energy demand MJ/d pReCiPe	Cradle-to-farm gate	1.32	—	4.13	8.48	0.10 ± 0.04	—	21.99 ± 3.84
	5.58 ± 2.07	OVO			1.51	—	4.01	8.92	0.12 ± 0.04	—	22.93 ± 6.40
	5.72 ± 2.28	PES			1.31	—	3.67	8.29	0.11 ± 0.04	—	22.29 ± 3.26
	5.05 ± 2.49	LME			2.02	—	5.29	10.02	0.17 ± 0.07	—	23.04 ± 3.79
	3.70 ± 2.66	MME			3.01	—	7.49	13.38	0.24 ± 0.09	—	24.15 ± 4.03
	0.57 ± 3.25	HME			4.79	—	12.06	18.80	0.38 ± 0.18	—	25.32 ± 4.68

Marty et al., 2022 [55] modified sPNNS-GS2 (-17 to 11.5)	1.2 ± 2.5	Total	GHGE kg CO_2_eq/d	Cradle-to-plate	4.8 ± 2.1	—	—	—	—	—	24.5 ± 4.9

Marty et al., 2022^3^ [70] modified sPNNS-GS2 (-17 to 11.5)	1.14 ± 2.49	Before	GHGE kg CO_2_eq/2000 kcal	Cradle-to-plate	5.67 ± 1.46	—	—	—	—	—	—
	0.88 ± 2.69	During			5.50 ± 1.40	—	—	—	—	—	24.38 ± 4.92
	1.15 ± 2.58	Mid-term			5.63 ± 1.45	—	—	—	—	—	24.67 ± 5.15

Kesse-Guyot et al., 2020^4^ [57] modified PNNS-GS1 (-17 to 13.5)	5.50	Q1	GHGE kg CO_2_eq/d Land occupation m^2^/d Energy demand MJ/d	Cradle-to-farm gate	4.24 (95% CI 4.18, 4.30)	—	10.84 (95% CI 10.67, 11.02)	16.78 (95% CI 16.62, 16.94)	0.33 (95% CI 0.33, 0.34)	—	24.25 ± 5.69
	7.29	Q2			4.19 (95% CI 4.13, 4.25)	—	10.84 (95% CI 10.68, 11.01)	17.14 (95% CI 16.98, 17.29)	0.33 (95% CI 0.32, 0.33)	—	24.23 ± 4.86
	8.18	Q3			4.01 (95% CI 3.95, 4.06)	—	10.45 (95% CI 10.30, 10.60)	17.16 (95% CI 17.01, 17.30)	0.30 (95% CI 0.30, 0.31)	—	24.14 ± 4.99
	9.09	Q4			4.00 (95% CI 3.95, 4.06)	—	10.45 (95% CI 10.30, 10.60)	17.78 (95% CI 17.64, 17.91)	0.28 (95% CI 0.28, 0.29)	—	24.53 ± 4.65
	10.49	Q5			3.78 (95% CI 3.73, 3.84)	—	9.89 (95% CI 9.75, 10.04)	17.95 (95% CI 17.82, 18.08)	0.25 (95% CI 0.25, 0.25)	—	24.45 ± 4.45

Kesse-Guyot et al., 2020^5^ [57] PNNS-GS2 (-17 to 14.25)	-3.13	Q1	GHGE kg CO_2_eq/d Land occupation m^2^/d Energy demand MJ/d	Cradle-to-farm gate	5.47 (95% CI 5.42, 5.53)	—	13.59 (95% CI 13.43, 13.75)	20.67 (95% CI 20.53, 20.82)	0.40 (95% CI 0.40, 0.41)	—	25.61 ± 0.06
	0.34	Q2			4.42 (95% CI 4.37, 4.48)	—	11.28 (95% CI 11.13, 11.43)	18.41 (95% CI 18.28, 18.55)	0.33 (95% CI 0.33, 0.34)	—	24.79 ± 0.06
	2.22	Q3			3.94 (95% CI 3.88, 3.99)	—	10.26 (95% CI 10.11, 10.41)	17.19 (95% CI 17.06, 17.32)	0.29 (95% CI 0.29, 0.30)	—	24.19 ± 0.06
	3.99	Q4			3.42 (95% CI 3.36, 3.47)	—	9.08 (95% CI 8.93, 9.23)	16.02 (95% CI 15.89, 16.16)	0.25 (95% CI 0.24, 0.25)	—	23.89 ± 0.06
	6.44	Q5			2.92 (95% CI 2.87,2.98)	—	8.14 (95% CI 7.98, 8.29)	14.84 (95% CI 14.71, 14.98)	0.20 (95% CI 0.20, 0.21)	—	23.18 ± 0.06

Baudry et al., 2019^6^ [71] modified PNNS-GS1 (-17 to 13.5)	8.12 (95% CI 8.10, 8.14)	Total	GHGE kg CO_2_eq/d Land occupation m^2^/d Energy demand MJ/d	Cradle-to-farm gate	4.48 (95% CI 4.44, 4.51)	-	11.58 (95% CI 11.49, 11.67)	18.61 (95% CI 18.51, 18.71)	-	-	24.95 (95% CI 24.88, 25.02)
	7.80 (95% CI 7.76, 7.84)	Q1			5.07 (95% CI 5.01, 5.12)	-	12.35 (95% CI 12.19, 12.51)	19.72 (95% CI 19.58, 19.85)	-	-	27.26 (95% CI 27.11, 27.41
	8.25 (95% CI 8.21, 8.29)	Q2			4.48 (95% CI 4.42, 4.53)	-	10.99 (95% CI 10.83, 11.15)	18.59 (95% CI 18.45, 18.73)	-	-	25.93 (95% CI 25.78, 26.08)
	8.31 (95% CI 8.27, 8.35)	Q3			4.48 (95% CI 4.43, 4.54)	-	11.40 (95% CI 11.24, 11.56)	18.45 (95% CI 18.31, 18.58)	-	-	25.13 (95% CI 24.98, 25.28)
	8.60 (95% CI 8.56, 8.64)	Q4			4.02 (95% CI 3.97, 4.08)	-	10.72 (95% CI 10.56, 10.87)	17.58 (95% CI 17.44, 17.72)	-	-	24.63 (95% CI 24.48, 24.78)
	8.83 (95% CI 8.79, 8.87)	Q5			3.17 (95% CI 3.12, 3.23)	-	9.52 (95% CI 9.36, 9.68)	14.67 (95% CI 14.54, 14.81)	-	-	23.36 (95% CI 23.21, 23.51)

Seconda et al., 2018^7^ [72] modified PNNS-GS1 (-17 to 13.5)	8.39 (95% CI 8.35, 8.43)	Q1	Land occupation m^2^/d∗ Energy demand MJ/d∗	Cradle-to-farm gate	-	-	4.64 (95% CI 4.42, 4.47)	10.90 (95% CI 9.81, 9.92)	-	-	23.52 (95% CI 23.38, 23.66)
	8.48 (95% CI 8.45, 8.52)	Q2			-	-	7.44 (95% CI 7.25, 7.32)	14.69 (95% CI 13.93, 14.08)	-	-	24.74 (95% CI 24.6, 24.87)
	8.64 (95% CI 8.61, 8.68)	Q3			-	-	9.94 (95% CI 9.89, 9.99)	17.22 (95% CI 17.14, 17.33)	-	-	26.01 (95% CI 25.87, 26.14)
	8.28 (95% CI 8.24, 8.32)	Q4			-	-	12.99 (95% CI 13.25, 13.40)	19.89 (95% CI 20.97, 21.22)	-	-	25.69 (95% CI 25.55, 25.83)
	7.94 (95% CI 7.89, 7.98)	Q5			-	-	19.69 (95% CI 20.90, 21.16)	24.60 (95% CI 28.52, 28.90)	-	-	26.59 (95% CI 26.43, 26.75)

Abbreviations: BMI, body mass index; GHGE, greenhouse gas emissions; HME: high-meat; LAC, lacto-vegetarian; LME, low-meat; MME, medium-meat; OVO, ovo-lacto-vegetarian; P, profile; PES, pesco-vegetarian; PNNS-GS1, Programme National Nutrition Santé – Guidelines Score 1; PNNS-GS2, Programme National Nutrition Santé – Guidelines Score 2; pReCiPe, partial ReCiPe; Q, quintile; sPNNS-GS2, Simplified Programme National Nutrition Santé – Guidelines Score 2.

Values are presented as mean; mean ± standard deviation (SD); mean & 95% confidence interval (CI)

1 Dietary protein consumption profiles: P1: Low meat: a low protein intake from meat (meat and poultry consumed 9 g versus 20.8 g/d total population); P2: Poultry: high intakes of protein from poultry (10.8 g versus 5.3 g/d total population); P3: Fish: highest fish protein intake (9.6 g versus 4.4 g/d in the total population); P4: Ruminant Meat: high intakes of protein from ruminant meat (12.4 g versus 6.1 g/d in the total population); P5: Pork: highest protein intake (9.6 g versus 2.7 g/d in the total population)

2 Diets with varying proportion of animal products: LAC <1 g/d of egg, meat, and seafood but consuming dairy products; OVO <1 g/d of meat and seafood but consuming eggs; PES <1 g/d of meat but consuming fish; LME total meat intake <50 g/d; MME: total meat intake 50–100 g/d; HME: total meat intake >100 g/d

3 Before first COVID-19 lockdown, during first lockdown (after 1 mo) and mid-term (1 y after first lockdown)

4 Sex-specific weighted quintiles of PNNS-GS1

5 Sex-specific weighted quintiles of PNNS-GS2

6 Weighted quintiles of proportion of organic food consumption in the diet: Q1 0% organic; Q2 4% organic; Q3 17% organic; Q4 34% organic; Q5 71% organic

7 Weighted quintiles according to diet-related GHGE (kg CO_2_eq/d); Q1 0.95–2.24; Q2 2.44–3.26; Q3 3.37–4.35; Q4 4.49–5.91; Q5 6.35–11.23

8 BMI was measured

9 Freshwater ecotoxicity in Comparative Toxic Unit for ecosystems (CTUe) an indicator based on a model called USEtox

10 System boundary as defined by study authors

∗ functional unit converted to daily impact for the purpose of comparison, originally reported by authors as per year

Conversely, Seconda et al. [72] compared the nutritional and environmental performance of diets according to quintiles of dietary GHGE. Participants in Q3 had the greatest compliance to the FBDG, although Q1 participants performed the best for reduced diet-related environmental impacts. The highest GHGE group (Q5) (6.35–11.23 kg CO_2_eq/d) had the lowest mPNNS-GS1 score and greater land occupation, and energy consumption compared to the Q1 group (0.95–2.24 kg CO_2_eq/d). The LO and energy demand increased with the level of dietary GHGE (adjusted for sex and age). With adjustment for EI, these associations remained, although the magnitudes of the differences were reduced.

For the PNNS-GS2 studies, Kesse-Guyot et al. [57] also categorized participants by level of adherence and reported lower environmental impact with increased adherence. For the pReCiPe score, a reduction of 50% was observed, with EI adjustment. The decreases observed in environmental pressure across quintiles were much greater with adherence to the 2017 FBDG than the 2001 recommendations. As the 2017 guidelines promoted the limitation of meat and moderation of dairy consumption, this may explain the lower levels of emissions in Q5 compared with Q1. Four studies did not report lower diet-related environmental impacts with greater guideline adherence [54,55,58,70]. The first, by Perraud et al. [58], characterized the population into 5 protein profiles, with those in the pork profile (P5) having the highest adherence. Energy use (9%) and freshwater ecotoxicity (8%) were the highest, and water use (3%) the second highest in P5 compared with the total population. GHGE were the same as the total population. Those in the low-meat profile (P1) had the lowest adherence (6%) compared with the total population. This profile had the lowest impact for most of the environmental indicators assessed (11 of 14) (not all shown in Table 4), and was the second-lowest for the remaining 3.

Kesse-Guyot et al. [54] compared 6 dietary groups and found that the pesco-vegetarian diet had the highest sPNNS-GS2 with the lowest in high-meat eaters. Pesco-vegetarians had the lowest environmental impacts across GHGE, land use, and energy demand, which was 73%, 70%, and 56% lower, respectively, than high-meat eaters. The high-meat diet was observed to be the worst for the environment. The authors optimized these dietary patterns under nutritional, epidemiological, co-production, acceptability, environmental, and cost constraints. Adherence to the guidelines for all dietary groups increased, specifically by 88% for pesco-vegetarians and 962% for high-meat eaters. Environmental pressures were reduced for both dietary groups under the optimized model.

Marty et al. [55] found that diet quality and environmental impact were not significantly associated with one another. Marty et al. [70] also performed a longitudinal analysis to examine the impacts on diet quality and environmental impacts before, during (after 1 mo), and mid-term (after 1 y) of the first COVID-19 lockdown. Diet quality decreased (23%) in the short-term, but no significant mid-term change was found. Dietary GHGE per 2000 kcal decreased (3%) in the short-term, but no significant sustained change was found. GHGE per day increased in the short-term then remained constant. There was little change in BMI after 1 y.

For BMI, 2 studies reported that greater guideline adherence resulted in lower BMI and environmental impacts [57,71], although when the same participants were stratified based on the 2001 FBDG, little difference in BMI was found [57]. One study found that BMI increased with GHGE, whereas adherence decreased [72]. In studies based on dietary pattern characteristics, high-meat eaters had the lowest adherence to the guidelines, but the highest environmental impacts and BMI [57], with the opposite found for low-meat eaters [58].

### Diet quality metric—Group B

#### Mediterranean Diet

Four studies reported compliance to the Mediterranean Diet (MD), each using a different metric [56,58,59,64]. Perraud et al. [58] calculated the Literature-Based Adherence Score to the Mediterranean Diet (LAMD) for 5 protein profiles within a French cohort. Those in the fish profile (P3), had the greatest adherence (11% higher than the total population), but contributed most to water use and ozone depletion (6% and 24% higher than the total population, respectively), and were the second highest emitter after those in the ruminant meat profile (Table 5). The mean BMI in this profile was the highest after the pork profile. The poultry profile (P2) had the lowest compliance to the MD (10% lower than the total population), but performed best across the environmental impact indicators after the low-meat profile.

**TABLE 5:** Mediterranean Diet Scores and the associated dietary environmental impacts and anthropometric markers

Author	Diet Quality		Environmental Impact					Ozone Depletion	Health Outcome	
	Adherence Score	Cohort Grouping	Functional Unit	System Boundary^7^	GHGE	Water	Land or Ecological		BMI kg/m^2^	%BF
Perraud et al., 2023^1^ [58] LAMD Score (0 to 18)	8.1 ± 2.8	Total	GHGE kg CO_2_eq/d Water m^3^/d Land use kg C deficit/d Ozone depletion^6^ Freon-11/d	Cradle-to-plate	6.4 ± 3.3	6.7 ± 3.2	314.4 ± 191.8	0.6 ± 1.1	24.9 ± 4.6^4^	—
	8.8 ± 2.9	P1			4.6 ± 1.9	6.0 ± 94.9	210.3 ± 7.4	0.4 ± 1.7	24.1 ± 4.4	—
	7.3 ± 2.6	P2			5.6 ± 2.6	6.2 ± 156.9	283.5 ± 9.0	0.6 ± 2.7	24.4 ± 4.5	—
	9.0 ± 2.8	P3			6.6 ± 3.4	7.1 ± 199.2	309.2 ± 11.4	0.7 ± 3.4	25.0 ± 4.7	—
	7.4 ± 2.7	P4			7.7 ± 4.5	5.9 ± 251.2	396.7 ± 13.6	0.5 ± 4.4	24.8 ± 5.0	—
	7.8 ± 2.5	P5			6.4 ± 2.9	6.9 ± 175.3	319.6 ± 14.2	0.6 ± 3.0	25.3 ± 4.4	—

Telleria-Aramburu et al., 2021^2^ [56] large-scale MDS (0 to 55)	33.53 ± 5.47	Total	GHGE kg CO_2_eq/d	Cradle-to-grave	4.71 (95% CI 4.69, 4.73)	—	—	—	22.3 (95% CI 22.3, 22.4)^4^	14.4^5^
	32.74 ± 5.20	Male			5.26 (95% CI 5.23, 5.30)	—	—	—	23.3 (95% CI 23.2, 23.3)	16.1
	34.07 ± 5.58	Female			4.34 (95% CI 4.31, 4.36)	—	—	—	21.7 (95% CI 21.6, 21.7)	13.3
	34.16 ± 6.41	Low-GHGE			2.84 (95% CI 2.83, 2.85)	—	—	—	21.6 (95% CI 21.5, 21.7)	7.0
	32.76 ± 5.57	High-GHGE			7.27 (95% CI 7.23, 7.32)	—	—	—	22.7 (95% CI 22.6, 22.8)	15.7

Murakami & Livingstone 2018^3^ [59] Non-Mediterranean populations MDS (0 to 9)	4.5 ± 1.7	Total	GHGE kg CO_2_eq/d	Cradle-to-grave	5.7 ± 2.1	—	—	—	27.4 ± 5.4^4^	—
	4.6 ± 1.7	Plausible reporters			6.3 ± 2.1	—	—	—	26.4 ± 4.9	—
	4.3 ± 1.7	Under- reporters			5.0 ± 1.8	—	—	—	28.6 ± 5.6	—

Rosi et al., 2017 [64] IMDI (0 to 11)	4.0 (3.0)	Omni	GHGE kg CO_2_eq/d∗ Water m^3^/d∗∗ Ecological footprint m^2^/d	Cradle-to-consumer waste	3.96 ± 0.98	3.14 ± 0.73	26.0 ± 5.6	—	22.1 ± 2.0^4^	—
	6.0 (2.0)	Ovo			2.60 ± 0.62	2.30 ± 0.42	16.1 ± 3.8	—	21.9 ± 2.5	—
	7.0 (2.0)	Veg			2.34 ± 0.50	2.46 ± 0.58	14.5 ± 3.1	—	21.3 ± 2.2	—

Abbreviations: BF, body fat; BMI, body mass index; EI:EER, ratio of energy intake estimated energy requirement; GHGE, greenhouse gas emissions; IMDI, Italian Mediterranean Diet Index; LAMD, Literature-Based Adherence Score to the Mediterranean Diet; MDS, Mediterranean Diet Score; Omni, omnivores; Ovo, ovo-lacto-vegetarians; P, profile; Veg, vegans.

Values are presented as mean ± standard deviation (SD); mean & 95% confidence interval (CI); median & interquartile range (IQR)

1 Dietary protein consumption profiles: P1: Low meat: a low protein intake from meat (meat and poultry consumed 9 g versus 20.8 g/d total population); P2: Poultry: high intakes of protein from poultry (10.8 g versus 5.3 g/d total population); P3: Fish: highest fish protein intake (9.6 g versus 4.4 g/d in the total population); P4: Ruminant meat: high intakes of protein from ruminant meat (12.4 g versus 6.1 g/d in the total population); P5: Pork: highest protein intake (9.6 g versus 2.7 g/d in the total population)

2 Low-GHGE diet (<3.39 kg CO_2_eq/d); High-GHGE diet (>5.78 kg CO_2_eq/d)

3 Plausible reporters (EI:EER 0.70–1.43); Under-reporters (EI:EER < 0.70)

4 BMI was measured

5 Values are percentage of participants classified as overweight/obese. Each participant’s BF was classified using the criteria proposed by Bray et al. (1998) [101] (body fat >33% and 25% for females and males, respectively). The %BF was calculated with skinfold data using the Siri-age-sex equation and the density estimated using the Durnin and Womersley formula

6 Ozone depletion potential in equivalent of kilograms of trichlorofluromethane (Freon-11)

7 System boundary as defined by study authors

∗ functional unit converted to kg for the purpose of comparison, originally reported by authors as g CO_2_eq/d

∗∗ functional unit converted to cubic meters (m^3^) for the purpose of comparison, originally reported by authors as L/d

Telleria-Aramburu et al. [56] measured adherence using a large-scale score in a Spanish cohort, and reported a score of 33.53 out of 55, with a difference between sexes found. Participants were ranked in quintiles of dietary GHGE per 1000 kcal/d. An inverse relationship was found between MD scores and GHGE, after controlling for sex, SES, and BF status. Participants with low-GHGE diets had a lower BMI and were less likely to have excessive BF compared to high-GHGE diets. Non-excessive adiposity was associated with higher diet quality scores.

Murakami and Livingstone [59] found that GHGE were not inversely associated with the MD score among a UK cohort, after adjustment for potential confounders (age, sex, ethnicity, SES, smoking status, and PAL). However, when further adjustment was made for the ratio of energy intake: estimated energy requirement (EI:EER), an inverse association between GHGE and MD score was found. For plausible reporters (EI:EER 0.70–1.43), an inverse association with MD adherence was observed. The inverse association for diet quality did not reach statistical significance among under-reporters (EI:EER < 0.70). Under-reporters had lower MD compliance (7%), GHGE (21%), and higher BMI (8%) compared with plausible reporters.

Rosi et al. [64] used the Italian Mediterranean Diet Index (IMDI) to compare 3 diet groups, and found that those following a vegan diet had higher MD adherence compared with omnivores and ovo-lacto-vegetarians. The omnivore group generated higher GHGE, water, and an EF, and had the lowest adherence. Subsequently, ovo-lacto-vegetarian and vegan diets had lower GHGE (34% and 41%), water (27% and 22%), and an EF (38% and 44%), respectively. Vegan and ovo-lacto-vegetarian diets showed a clear environmental and diet quality advantage with respect to the omnivore diet. However, no significant difference between vegans and ovo-lacto-vegetarians was found. The authors hypothesized that this may be a result of higher food intake among vegans with respect to ovo-lacto-vegetarians (∼12.5% in terms of food weight), as PB foods have lower energy density. BMI was similar for the 3 groups.

### Diet quality metric—Group C

#### Alternative Healthy Eating Index

Three studies [53,58,73] measured diet quality using the Alternative Healthy Eating Index 2010 (AHEI-2010). Perraud et al. [58] reported that those in the fish profile (P3) had the highest score (7% higher than the total population), but performed the worst for 4 of the 14 indicators (ozone depletion, photochemical ozone, freshwater eutrophication, and water use) (Table 6). The ruminant meat profile (P4) had the lowest compliance (8% lower than the total population). This profile had the highest environmental impact for 5 of the 14 indicators assessed (not all shown in Table 6). This included GHGE, land use, the emission of particulate matter, acidification, and terrestrial eutrophication, which were 20%, 26%, 14%, 16%, and 19% higher, respectively, than the total population. BMI was slightly lower in the P4 profile than P3. Frehner et al. [73] reported lower AHEI scores in a Swiss cohort. Dietary GHGE and grassland occupation (GLO) were not significantly different between males and females. Being male was negatively associated with the AHEI, and positively associated with cropland occupation. Overweight was positively associated with GLO, and overweight and obesity negatively associated with diet quality.

**TABLE 6:** AHEI, DASH, and HDI adherence with the associated dietary environmental impacts, BMI, and blood pressure

Author	Diet quality		Environmental impact							Health outcome		
	Adherence score	Cohort grouping	Functional unit	System boundary^8^	GHGE	Land	Eutrophication	Eutrophication (terrestrial)	Acidification	BMI kg/m^2^	SBP mmHg	DBP mmHg
**Alternative Healthy Eating Index (AHEI)**												
Perraud et al., 2023^1^ [58] modified (0 to 100)	43.4 ± 12.2	Total	GHGE kg CO_2_eq/d Land kg C deficit/d Eutrophication (freshwater) kg Peq/d Eutrophication (terrestrial) mol Neq/d Acidification mol H^+^eq/d	Cradle-to-plate	6.4 ± 3.3	314.4 ± 191.8	24.5 ± 12.2	0.3 ± 0.2	0.1 ± 0.1	24.9 ± 4.6^7^	—	—
	43.9 ± 11.6	P1			4.6 ± 1.9	210.3 ± 7.4	19.2 ± 0.1	0.2 ± 0.2	0.1 ± 8.2	24.1 ± 4.4	—	—
	42.3 ± 13.6	P2			5.6 ± 2.6	283.5 ± 9.0	21.1 ± 0.2	0.3 ± 0.3	0.1 ± 5.8	24.4 ± 4.5	—	—
	46.5 ± 12.2	P3			6.6 ± 3.4	309.2 ± 11.4	25.2 ± 0.2	0.3 ± 0.3	0.1 ± 10.9	25 ± 4.7	—	—
	40.0 ± 10.6	P4			7.7 ± 4.5	396.7 ± 13.6	25.9 ± 0.3	0.4 ± 0.4	0.1 ± 9.4	24.8 ± 5.0	—	—
	42.6 ± 11.3	P5			6.4 ± 2.9	319.6 ± 14.2	26.0 ± 0.2	0.3 ± 0.3	0.1 ± 9.7	25.3 ± 4.4	—	—

Frehner et al., 2021 [73] (0 to 110)	43.65	Total	GHGE kg CO_2_eq/d Land occupation m^2^/d	Cradle-to-point of retail	3.25	6.35	—	—	—	25.0 ± 4.4^7^	—	—
	43.00	Male			3.26	6.42	—	—	—	25.9 ± 3.9	—	—
	44.30	Female			3.25	6.28	—	—	—	24.0 ± 4.7	—	—

Hobbs et al., 2020^2^ [53] (0 to 110)	56 (95% CI 55, 56)^5^	Total	GHGE kg CO_2_eq/d Eutrophication g Neq/d Acidification g SO_2_eq/d	Cradle-to-point of retail	4.1 (95% CI 4.0, 4.1)^5^	—	54.0 (95% CI 52.3, 55.7)^5^	—	35.2 (95% CI 34.4, 36.0)^5^	-	—	—
	53 (95% CI 52, 54)^5^	Qu1			4.0 (95% CI 3.9, 4.1)	—	50.8 (95% CI 47.7, 54.0)	—	35.9 (95% CI 34.5, 37.3)	28^7^ (95% CI 27, 29)^5^^,^^7^	126 (95% CI 124, 129)^5^	76 (95% CI 74, 78)^5^
	56 (95% CI 55, 57)^5^	Qu2			4.1 (95% CI 4.0, 4.2)	—	51.9 (95% CI 48.7, 55.0)	—	34.4 (95% CI 33.1, 35.8)	28 (95% CI 27, 29)^5^	125 (95% CI 122, 127)^5^	75 (95% CI 73, 76)^5^
	57 (95% CI 55, 58)^5^	Qu3			4.0 (95% CI 3.9, 4.1)	—	52.5 (95% CI 49.4, 55.6)	—	34.2 (95% CI 32.9, 35.6)	27 (95% CI 26, 28)^5^	126 (95% CI 123, 128)^5^	76 (95% CI 74, 77)^5^
	58 (95% CI 57, 59)^5^	Qu4			4.1 (95% CI 4.0, 4.2)	—	60.7 (95% CI 57.5, 63.9)	—	36.1 (95% CI 34.8, 37.5)	27 (95% CI 26, 28)^5^	124 (95% CI 122, 126)^5^	73 (95% CI 72, 75)^5^

**Dietary Approaches to Stop Hypertension (DASH)**												
Murakami & Livingstone 2018^3^ [59] modified (8 to 40)	24.3 ± 5.2	Total	GHGE kg CO_2_eq/d	Cradle-to-grave	5.7 ± 2.1	—	—	—	—	27.4 ± 5.4^7^	—	—
	23.9 ± 5.2	Plausible reporters			6.3 ± 2.1	—	—	—	—	26.4 ± 4.9	—	—
	24.7 ± 5.2	Under- reporters			5.0 ± 1.8	—	—	—	—	28.6 ± 5.6	—	—

Biesbroek et al., 2017^4^ [60] (8 to 40)	24.0 ± 4.8	Male	GHGE kg CO_2_eq/d Land m^2^×y/d	Cradle-to-grave	4.6 ± 0.1	4.4 ± 0.1	—	—	—	25.6 ± 3.2^7^	—	—
	24.0 ± 4.9	Female			3.7 ± 0.1	3.50 ± 0.1	—	—	—	25.0 ± 3.7	—	—
	19.2 ± 2.5	Male T1			4.59 ± 0.1	4.42 ± 0.1	—	—	—	25.9 ± 3.8	—	—
	24.5 ± 1.1	Male T2			4.64 ± 0.1	4.39 ± 0.1	—	—	—	25.7 ± 3.4	—	—
	29.6 ± 2.4	Male T3			4.62 ± 0.1	4.30 ± 0.1	—	—	—	25.4 ± 3.4	—	—
	19.0 ± 2.6	Female T1			3.68 ± 0.1	3.49 ± 0.1	—	—	—	25.7 ± 4.3	—	—
	24.5 ± 1.1	Female T2			3.78 ± 0.1	3.49 ± 0.1	—	—	—	25.6 ± 4.0	—	—
	29.5 ± 2.3	Female T3			3.77 ± 0.1	3.36 ± 0.1	—	—	—	25.3 ± 4.0	—	—

**Healthy Diet Indicator (HDI)**												
Murakami & Livingstone 2018^3^ [59] (0 to 7)	2.3 ± 1.1	Total	GHGE kg CO_2_eq/d	Cradle-to-grave	5.7 ± 2.1	—	—	—	—	27.4 ± 5.4^7^	—	—
	2.2 ± 1.2	Plausible reporters			6.3 ± 2.1	—	—	—	—	26.4 ± 4.9	—	—
	2.4 ± 1.1	Under- reporters			5.0 ± 1.8	—	—	—	—	28.6 ± 5.6	—	—

Biesbroek et al., 2017^6^ [60] (0 to 7)	3.3 ± 1.2	Male	GHGE kg CO_2_eq/d Land m^2^×y/d	Cradle-to-grave	4.6 ± 0.1	4.4 ± 0.1	—	—	—	25.6 ± 3.2^7^	—	—
	3.3 ± 1.3	Female			3.7 ± 0.1	3.5 ± 0.1	—	—	—	25.0 ± 3.7	—	—
	1.8 ± 0.5	Male C1			4.87 ± 0.1	4.56 ± 0.1	—	—	—	26.0 ± 3.5	—	—
	3.0 ± 0.0	Male C2			4.66 ± 0.1	4.42 ± 0.1	—	—	—	25.8 ± 3.5	—	—
	4.4 ± 0.6	Male C3			4.42 ± 0.1	4.21 ± 0.1	—	—	—	25.5 ± 3.4	—	—
	1.9 ± 0.4	Female C1			3.83 ± 0.1	3.53 ± 0.1	—	—	—	25.6 ± 4.2	—	—
	3.0 ± 0.0	Female C2			3.75 ± 0.1	3.50 ± 0.1	—	—	—	25.6 ± 4.1	—	—
	4.5 ± 0.6	Female C3			3.66 ± 0.1	3.35 ± 0.1	—	—	—	25.4 ± 4.0	—	—

Abbreviations: BMI, body mass index; DBP, diastolic blood pressure; EI:EER, ratio of energy intake estimated energy requirement; GHGE, greenhouse gas emissions. P, profile; Qu, quartile; SBP, systolic blood pressure; T, tertile.

Values are presented as mean ± standard deviation (SD); median; mean & 95% confidence interval (CI)

1 Dietary protein consumption profiles: P1: Low meat: a low protein intake from meat (meat and poultry consumed 9 g versus 20.8 g/d total population); P2: Poultry: high intakes of protein from poultry (10.8 g versus 5.3 g/d total population); P3: Fish: highest fish protein intake (9.6 g versus 4.4 g/d in the total population); P4: Ruminant meat: high intakes of protein from ruminant meat (12.4 g versus 6.1 g/d in the total population); P5: Pork: highest protein intake (9.6 g versus 2.7 g/d in the total population)

2 Quartiles of total dairy product consumption (milk, cheese, yogurt, dairy desserts): Q1: 0–96 g/d; Q2: 97–172 g/d; Q3: 173–273 g/d; Q4: 274–1429 g/d

3 Plausible reporters (EI:EER 0.70–1.43); Under-reporters (EI:EER < 0.70)

4 Tertiles of Dietary Approaches to Stop Hypertension (DASH) adherence (Males: T1 ≤22; T2 23–26; T3 ≥27; Females: T1 ≤22; T2 23–26; T3 ≥27)

5 Value is non-adjusted mean (95% CIs)

6 Categories of Healthy Diet Indicator (HDI) adherence (Males: C1 0-2; C2 3; C3 4-7; Females: C1 0-2; C2 3; C3 4-7)

7 BMI was measured

8 System boundary as defined by study authors

Hobbs et al. [53] stratified a UK population into quartiles of total dairy product consumption, with those in Q2, Q3, and Q4 having higher scores compared with lower dairy consumption diets (Q1). Diets containing the highest amount of dairy (Q4) had higher eutrophication potential (19%) compared with Q1 diets (adjusted for age, sex, and EI). GHGE and acidification potential were also higher but in the non-adjusted model only. For cardiometabolic risk factors, systolic blood pressure and diastolic blood pressure were different across quartiles of total dairy intake (adjusted for age, sex, BMI and EI). For BMI, total cholesterol, LDL and HDL cholesterol, and others, there were no significant differences.

#### Dietary Approaches to Stop Hypertension diet

Two studies [59,60] measured compliance with the DASH diet. Murakami and Livingstone [59] reported moderate adherence to DASH in the United Kingdom. Dietary GHGE were inversely associated with the DASH score after adjustment for potential confounding factors (discussed previously). With adjustment for EI:EER, GHGE remained inversely associated with DASH. For under-reporters only, an inverse association was observed irrespective of adjustment. Similar associations were also observed when plausible reporters were analyzed separately. The under-reporters had higher DASH scores (3%) compared with plausible reporters.

Similarly, Biesbroek et al. [60] found moderate adherence for both males and females in the Netherlands (Table 6). Participants were ranked across sex-specific tertiles of DASH adherence. When comparing T3 with T1, land use was 3% lower in males, but GHGE increased by 1% (not significant), after adjustments (discussed previously). Higher scores on the DASH diet (T3) were associated with higher GHGE (2%) and lower land use (4%) among females. BMI was consistently lower with higher diet quality scores.

#### Comprehensive Diet Quality Index

Kesse-Guyot et al. [54] assessed 6 dietary groups using the comprehensive Diet Quality Index (cDQI). Those following a pesco-vegetarian diet had the highest score (54.98 out of 85), whereas the lowest was found in those with a high-meat diet (45.40). The pesco-vegetarians and high-meat eaters had the lowest and highest environmental impacts, respectively. Under an optimized model, considering various constraints (outlined previously), all dietary groups increased their cDQI score, specifically a 15% improvement for pesco-vegetarians and 35% for high-meat eaters. Reductions in environmental impacts (GHGE, land use, and energy) were observed for both groups.

#### Health Diet Indicator

Two studies [59,60] measured adherence to the 2002 WHO’s guidelines for prevention of chronic diseases, using the Health Diet Indicator (HDI). Biesbroek et al. [60] reported low adherence for both Dutch males and females (Table 6). GHGE and land use were lower for males and females (9% and 4%; 8% and 5%, respectively), when comparing the highest adherence with the lowest after adjustments (detailed previously). Murakami and Livingstone [59] reported lower adherence to the HDI in the United Kingdom, and dietary GHGE were inversely associated with HDI scores, after adjustment for potential confounders (detailed previously). With further adjustments for EI:EER, the same associations were observed. Similar to the total population, inverse associations were observed when under- and plausible reporters were analyzed separately, and irrespective of adjustments. The under-reporters had higher HDI scores (9%) compared with plausible reporters.

### Diet quality metric—Group D

#### EAT-Lancet Diet

Three studies (55,62,74) assessed compliance with the EAT-Lancet Diet (ELD). With a cohort from ten European countries (see Table 2), Laine et al. [74] reported a mean adherence score of 8 out of 14. Those with greater adherence (13 points) had lower environmental pressures compared with those with the least adherence (3 points) (Table 7). As such, an increase of 10 points could result in a 50% and 62% reduction in GHGE and land use, respectively. The majority of the cohort were either overweight or obese (mean BMI 25 kg/m^2^). Similarly, Marty et al. [55] found that adherence to the diet was negatively associated with GHGE, based on the EAT-Lancet Diet Index (ELD-I). The population mean BMI was 24.5 kg/m^2^. Kesse-Guyot et al. [62] ranked participants into quintiles, reflecting the level of adherence to the ELD-I. As with the other studies, negative associations were observed between the ELD and environmental indicators. Greater adherence (Q5: >59.74 points) was associated with lower GHGE, CED, and LO compared with low adherence (Q1: ≤4.35), a reduction of 53%, 26%, and 50%, respectively, after adjustment for EI. For the pReCipe score, a reduction of 61% was observed between Q5 and Q1. The authors highlighted that the pReCipe score, despite lowering across quintiles, showed great variability, especially in Q1. Similar findings were found for the individual environmental indicators also. Those with greater adherence had lower BMI compared with least-adherent participants (mean BMI about -2 kg/m^2^).

**TABLE 7:** Eat Lancet Diet adherence and the associated dietary environmental impacts and body mass index

Author	Diet quality		Environmental impact						Health outcome
	Adherence score	Cohort grouping	Functional unit	System boundary^4^	GHGE	Land	Energy	pReCiPe score	BMI kg/m^2^
Marty et al., 2022 [55] ELD-I (continuous)^1^	-16.0 ± 37.1	Total	GHGE kg CO_2_eq/d	Cradle-to-plate	4.8 ± 2.1	—	—	—	24.5 ± 4.9

Kesse-Guyot et al., 2021 [62] ELD-I (continuous)^1^^,^^2^	-13.24 ± 16.46	Q1	GHGE kg CO_2_eq/d Land use m^2^/d Energy demand MJ/d pReCiPe	Cradle-to-farm gate	5.83 (95% CI 5.79, 5.88)	14.99 (95% CI 14.86, 15.12)	21.18 (95% CI 21.05, 21.30)	0.44 (95% CI 0.44, 0.45)	25.12 ± 4.95
	13.29 ± 4.87	Q2			4.44 (95% CI 4.40, 4.49)	11.48 (95% CI 11.35, 11.61)	18.13 (95% CI 18.00, 18.25)	0.33 (95% CI 0.32, 0.33)	24.62 ± 4.62
	29.38 ± 4.64	Q3			3.88 (95% CI 3.84, 3.93)	10.14 (95% CI 10.01, 10.27)	17.10 (95% CI 16.97, 17.23)	0.27 (95% CI 0.27, 0.28)	24.32 ± 4.65
	47.81 ± 6.33	Q4			3.38 (95% CI 3.33, 3.42)	8.96 (95% CI 8.83, 9.09)	16.16 (95% CI 16.04, 16.29)	0.23 (95% CI 0.23, 0.23)	23.82 ± 4.44
	88.85 ± 31.02	Q5			2.73 (95% CI 2.69, 2.78)	7.45 (95% CI 7.32, 7.58)	15.58 (95% CI 15.45, 15.71)	0.17 (95% CI 0.17, 0.17)	23.13 ± 4.21

Laine et al., 2021 [74] ELD score (0 to 14)	8 (3–13)	Total	GHGE kg CO_2_eq/d Land use m^2^×year/d	Cradle-to-plate	6.0 (0.68–30.10)	7.2 (0.79–48.40)	—	—	25 (10–78)^3^

Abbreviations: ELD, Eat Lancet Diet; ELD-I, Eat Lancet Diet Index; GHGE, greenhouse gas emissions; pReCiPe, partial ReCiPe; Q, quintile.

Values are presented as mean ± standard deviation (SD); Mean & 95% confidence interval (CI); mean & (range)

1 A diet that meets the ELD recommendations is zero

2 Quintiles of Eat Lancet Diet Index (ELD-I); Q1 ≤4.35; Q2 4.35-21.46; Q3 21.46-37.67; Q4 37.67-59.74; Q5 >59.74

3 BMI was both measured and self-reported

4 System boundary as defined by study authors

### Affordability

Seven studies [53,54,57,62,[71], [72], [73]] reported on dietary cost for the following diet quality metrics, AHEI (*n*=2), cDQI, ELD-I, PNNS-GS1 (*n*=3), and PNNS-GS2 (*n*=2). Hobbs et al. [53] found that diets with higher AHEI scores and dairy consumption had a lower financial cost (-19%) (adjustments previously detailed). Frehner et al. [73] reported lower AHEI adherence but a higher dietary cost.

Three studies [57,62,71] reported that higher diet quality increased cost. This was observed with greater adherence to the ELD-I, with a 10% higher dietary cost (Table 8). However, the authors noted that the association appeared to be J-shaped [62]. Baudry et al. [71] found that adherence to the 2001 FBDG were highest in those with the greatest proportion of organic food consumption (Q5), and resulted in higher costs (26%) compared with those who do not consume organic food (Q1) (adjustments outlined previously). Kesse-Guyot et al. [57] assessed compliance with both the 2001 and 2017 guidelines. The cost of the diet was positively associated with PNNS-GS1 and PNNS-GS2. However, the size of the increase between Q1 (low adherence) and Q5 (high adherence) was smaller for PNNS-GS2. The differences between Q5 and Q1 were €0.91 and €1.29 per day for the PNNS-GS2 and PNNS-GS1, respectively.

**TABLE 8:** Studies reporting dietary cost of adherence to diet quality metrics and associated dietary environmental impacts and body mass index

Author	Diet quality		Functional Unit	Environmental impact							Health outcome	Affordability
	Adherence Score	Cohort grouping		System boundary^10^	GHGE	Land	Energy	Acidification	Eutrophication	pReCiPe	BMI kg/m^2^	Price €/d
Kesse-Guyot et al., 2022^1^ [54] sPNNS-GS2 (-17 to 14.25)	5.64 ± 1.97	LAC	GHGE kg CO_2_eq/d Land occupation m^2^/d Energy demand MJ/d pReCiPe	Cradle-to-farm gate	1.32	4.13	8.48	—	—	0.10 ± 0.04	21.99 ± 3.84	9.04 ± 4.98
	5.58 ± 2.07	OVO			1.51	4.01	8.92	—	—	0.12 ± 0.04	22.93 ± 6.40	8.09 ± 3.81
	5.72 ± 2.28	PES			1.31	3.67	8.29	—	—	0.11 ± 0.04	22.29 ± 3.26	8.94 ± 4.77
	5.05 ± 2.49	LME			2.02	5.29	10.02	—	—	0.17 ± 0.07	23.04 ± 3.79	6.70 ± 2.89
	3.70 ± 2.66	MME			3.01	7.49	13.38	—	—	0.24 ± 0.09	24.15 ± 4.03	6.95 ± 2.50
	0.57 ± 3.25	HME			4.79	12.06	18.80	—	—	0.38 ± 0.18	25.32 ± 4.68	8.77 ± 2.91

Kesse-Guyot et al., 2022^1^ [54] cDQI (0 to 85)	47.06 ± 6.13	LAC	GHGE kg CO_2_eq/d Land occupation m^2^/d Energy demand MJ/d pReCiPe	Cradle-to-farm gate	1.32	4.13	8.48	—	—	0.10 ± 0.04	21.99 ± 3.84	9.04 ± 4.98
	50.03 ± 6.68	OVO			1.51	4.01	8.92	—	—	0.12 ± 0.04	22.93 ± 6.40	8.09 ± 3.81
	54.98 ± 7.42	PES			1.31	3.67	8.29	—	—	0.11 ± 0.04	22.29 ± 3.26	8.94 ± 4.77
	51.71 ± 8.25	LME			2.02	5.29	10.02	—	—	0.17 ± 0.07	23.04 ± 3.79	6.70 ± 2.89
	48.28 ± 8.68	MME			3.01	7.49	13.38	—	—	0.24 ± 0.09	24.15 ± 4.03	6.95 ± 2.50
	45.40 ± 8.36	HME			4.79	12.06	18.80	—	—	0.38 ± 0.18	25.32 ± 4.68	8.77 ± 2.91

Frehneret al., 2021 [73] AHEI (0 to 110)	43.65	Total	GHGE kg CO_2_eq/d Land occupation m^2^/d	Cradle-to-point of retail	3.25	6.35	—	—	—	—	25.0 ± 4.4^8^	9.70∗
	43.00	Male			3.26	6.42	—	—	—	—	25.9 ± 3.9	—
	44.30	Female			3.25	6.28	—	—	—	—	24.0 ± 4.7	—

Kesse-Guyot et al., 2021^2^ [62] ELD-I (continuous)	-13.24 ± 16.46	Q1	GHGE kg CO_2_eq/d Land occupation m^2^/d Energy demand MJ/d	Cradle-to-farm gate	5.83 (95% CI 5.79, 5.88)	14.99 (95% CI 14.86, 15.12)	21.18 (95% CI 21.05, 21.30)	—	—	0.44 (95% CI 0.44, 0.45)	25.12 ± 4.95	7.72 ± 2.92
	13.29 ± 4.87	Q2			4.44 (95% CI 4.40, 4.49)	11.48 (95% CI 11.35, 11.61)	18.13 (95% CI 18.00, 18.25)	—	—	0.33 (95% CI 0.32, 0.33)	24.62 ± 4.62	7.38 ± 2.65
	29.38 ± 4.64	Q3			3.88 (95% CI 3.84, 3.93)	10.14 (95% CI 10.01, 10.27)	17.10 (95% CI 16.97, 17.23)	—	—	0.27 (95% CI 0.27, 0.28)	24.32 ± 4.65	7.43 ± 2.73
	47.81 ± 6.33	Q4			3.38 (95% CI 3.33, 3.42)	8.96 (95% CI 8.83, 9.09)	16.16 (95% CI 16.04, 16.29)	—	—	0.23 (95% CI 0.23, 0.23)	23.82 ± 4.44	7.48 ± 2.73
	88.85 ± 31.02	Q5			2.73 (95% CI 2.69, 2.78)	7.45 (95% CI 7.32, 7.58)	15.58 (95% CI 15.45, 15.71)	—	—	0.17 (95% CI 0.17, 0.17)	23.13 ± 4.21	8.53 ± 3.66

Hobbs et al., 2020^3^ [53] AHEI (0 to 110)	56 (95% CI 55, 56)^9^	Total	GHGE kg CO_2_eq/d Eutrophication g Neq/d Acidification g SO_2_eq/d	Cradle-to-point of retail	4.1 (95% CI 4.0, 4.1)^9^	—	—	54.0 (95% CI 52.3, 55.7)^9^	35.2 (95% CI 34.4, 36.0)^9^	—	—	6.17 (95% CI 6.06, 6.29)^9^^,^∗∗
	53 (95% CI 52, 54)^9^	Qu1			4.0 (95% CI 3.9, 4.1)	—	—	50.8 (95% CI 47.7, 54.0)	35.9 (95% CI 34.5, 37.3)	—	28 (95% CI 27, 29)^9^	6.75 (95% CI 6.52, 6.87)∗∗
	56 (95% CI 55, 57)^9^	Qu2			4.1 (95% CI 4.0, 4.2)	—	—	51.9 (95% CI 48.7, 55.0)	34.4 (95% CI 33.1, 35.8)	—	28 (95% CI 27, 29)^9^	6.52 (95% CI 6.29, 6.75)∗∗
	57 (95% CI 55, 58)^9^	Qu3			4.0 (95% CI 3.9, 4.1)	—	—	52.5 (95% CI 49.4, 55.6)	34.2 (95% CI 32.9, 35.6)	—	27 (95% CI 26, 28)^9^	5.94 (95% CI 5.71, 6.06)∗∗
	58 (95% CI 57, 59)^9^	Qu4			4.1 (95% CI 4.0, 4.2)	—	—	60.7 (95% CI 57.5, 63.9)	36.1 (95% CI 34.8, 37.5)	—	27 (95% CI 26, 28)^9^	5.47 (95% CI 5.36, 5.71)∗∗

Kesse-Guyot et al. 2020^4^ [57] modified PNNS-GS1 (-17 to 13.5)	5.5	Q1	GHGE kg CO_2_eq/d Land occupation m^2^/d Energy demand MJ/d	Cradle-to-farm gate	4.24 (95% CI 4.18, 4.30)	10.84 (95% CI 10.67, 11.02)	16.78 (95% CI 16.62, 16.94)	—	—	0.33 (95% CI 0.33, 0.34)	24.25 ± 5.69	6.83 (95% CI 6.77, 6.89)
	7.28	Q2			4.19 (95% CI 4.13, 4.25)	10.84 (95% CI 10.68, 11.01)	17.14 (95% CI 16.98, 17.29)	—	—	0.33 (95% CI 0.32, 0.33)	24.23 ± 4.86	7.06 (95% CI 7.00, 7.12)
	8.18	Q3			4.01 (95% CI 3.95, 4.06)	10.45 (95% CI 10.30, 10.60)	17.16 (95% CI 17.01, 17.30)	—	—	0.30 (95% CI 0.30, 0.31)	24.14 ± 4.99	7.28 (95% CI 7.23, 7.34)
	9.09	Q4			4.00 (95% CI 3.95, 4.06)	10.45 (95% CI 10.30, 10.60)	17.78 (95% CI 17.64, 17.91)	—	—	0.28 (95% CI 0.28, 0.29)	24.53 ± 4.65	7.68 (95% CI 7.63, 7.74)
	10.49	Q5			3.78 (95% CI 3.73, 3.84)	9.89 (95% CI 9.75, 10.04)	17.95 (95% CI 17.82, 18.08)	—	—	0.25 (95% CI 0.25, 0.25)	24.45 ± 4.45	8.12 (95% CI 8.07, 8.17)

Kesse-Guyot et al. 2020^5^ [57] PNNS-GS2 (-17 to 14.25)	-3.13	Q1	GHGE kg CO_2_eq/d Land occupation m^2^/d Energy demand MJ/d	Cradle-to-farm gate	5.47 (95% CI 5.42, 5.53)	13.59 (95% CI 13.43, 13.75)	20.67 (95% CI 20.53, 20.82)	—	—	0.40 (95% CI 0.40, 0.41)	25.61 ± 0.06	7.07 (95% CI 7.01, 7.13)
	0.34	Q2			4.42 (95% CI 4.37, 4.48)	11.28 (95% CI 11.13, 11.43)	18.41 (95% CI 18.28, 18.55)	—	—	0.33 (95% CI 0.33, 0.34)	24.79 ± 0.06	7.24 (95% CI 7.18, 7.29)
	2.22	Q3			3.94 (95% CI 3.88, 3.99)	10.26 (95% CI 10.11, 10.41)	17.19 (95% CI 17.06, 17.32)	—	—	0.29 (95% CI 0.29, 0.30)	24.19 ± 0.06	7.36 (95% CI 7.31, 7.41)
	3.99	Q4			3.42 (95% CI 3.36, 3.47)	9.08 (95% CI 8.93, 9.23)	16.02 (95% CI 15.89, 16.16)	—	—	0.25 (95% CI 0.24, 0.25)	23.89 ± 0.06	7.58 (95% CI 7.52, 7.63)
	6.44	Q5			2.92 (95% CI 2.87, 2.98)	8.14 (95% CI 7.98, 8.29)	14.84 (95% CI 14.71, 14.98)	—	—	0.20 (95% CI 0.20, 0.21)	23.18 ± 0.06	7.98 (95% CI 7.92, 8.03)

Baudry et al., 2019^6^ [71] modified PNNS-GS1 (-17 to 13.5)	8.12 (95% CI 8.10, 8.14)	Total	GHGE kg CO_2_eq/d Land occupation m^2^/d Energy demand MJ/d	Cradle-to-farm gate	4.48 (95% CI 4.44, 4.51)	11.58 (95% CI 11.49, 11.67)	18.61 (95% CI 18.51, 18.71)	—	—	—	24.95 (95% CI 24.88, 25.02)	—
	7.80 (95% CI 7.76, 7.84)	Q1			5.07 (95% CI 5.01, 5.12)	12.35 (95% CI 12.19, 12.51)	19.72 (95% CI 19.58, 19.85)	—	—	—	27.26 (95% CI 27.11, 27.41	7.11 (95% CI 7.03, 7.18)
	8.25 (95% CI 8.21, 8.29)	Q2			4.48 (95% CI 4.42, 4.53)	10.99 (95% CI 10.83, 11.15)	18.59 (95% CI 18.45, 18.73)	—	—	—	25.93 (95% CI 25.78, 26.08)	7.48 (95% CI 7.41, 7.55)
	8.31 (95% CI 8.27, 8.35)	Q3			4.48 (95% CI 4.43, 4.54)	11.40 (95% CI 11.24, 11.56)	18.45 (95% CI 18.31, 18.58)	—	—	—	25.13 (95% CI 24.98, 25.28)	7.77 (95% CI 7.70, 7.85)
	8.60 (95% CI 8.56, 8.64)	Q4			4.02 (95% CI 3.97, 4.08)	10.72 (95% CI 10.56, 10.87)	17.58 (95% CI 17.44, 17.72)	—	—	—	24.63 (95% CI 24.48, 24.78)	8.19 (95% CI 8.11, 8.26)
	8.83 (95% CI 8.79, 8.87)	Q5			3.17 (95% CI 3.12, 3.23)	9.52 (95% CI 9.36, 9.68)	14.67 (95% CI 14.54, 14.81)	—	—	—	23.36 (95% CI 23.21, 23.51)	8.97 (95% CI 8.90, 9.05)

Seconda et al., 2018^7^ [72] modified PNNS-GS1 (-17 to 13.5)	8.39 (95% CI 8.35, 8.43)	Q1	Land occupation m^2^/d∗∗∗ Energy demand MJ/d∗∗∗	Cradle-to-farm gate	—	4.64 (95% CI 4.42, 4.47)	10.90 (95% CI 9.81, 9.92)	—	—	—	23.52 (95% CI 23.38, 23.66)	6.89 (95% CI 6.84, 6.93)
	8.48 (95% CI 8.45, 8.52)	Q2			—	7.44 (95% CI 7.25, 7.32)	14.69 (95% CI 13.93, 14.08)	—	—	—	24.74 (95% CI -24.6, 24.87)	6.99 (95% CI 6.95, 7.03)
	8.64 (95% CI 8.61, 8.68)	Q3			—	9.94 (95% CI 9.89, 9.99)	17.22 (95% CI 17.14, 17.33)	—	—	—	26.01 (95% CI 25.87, 26.14)	7.20 (95% CI 7.16, 7.25)
	8.28 (95% CI 8.24, 8.32)	Q4			—	12.99 (95% CI 13.25, 13.40)	19.89 (95% CI 20.97, 21.22)	—	—	—	25.69 (95% CI 25.55, 25.83)	7.47 (95% CI 7.42, 7.52)
	7.94 (95% CI 7.89, 7.98)	Q5			—	19.69 (95% CI 20.90, 21.16)	24.60 (95% CI 28.52, 28.90)	—	—	—	26.59 (95% CI 26.43, 26.75)	7.68 (95% CI 7.62, 7.74)

Abbreviations: AHEI, Alternative Healthy Eating Index; BMI, body mass index; cDQI, Comprehensive Diet Quality Index; ELD-I, Eat Lancet Diet Index; GHGE, greenhouse gas emissions; HME: high-meat; LAC, lacto-vegetarian; LME, low-meat; MME, medium-meat; OVO, ovo-lacto-vegetarian; PES, pesco-vegetarian; PNNS-GS1, Programme National Nutrition Santé – Guidelines Score 1; PNNS-GS2, Programme National Nutrition Santé – Guidelines Score 2; pReCiPe, partial ReCiPe; Q, quintile; Qu, quartile; sPNNS-GS2, Simplified Programme National Nutrition Santé – Guidelines Score 2.

Values are presented as mean (Kesse-Guyot et al. 2022 [54]); mean ± standard deviation (SD); median (Frehner et al. 2021 [83]); mean & 95% confidence interval (CI)

Currency conversion from the following website https://www.xe.com/ (accessed 30 January, 2023).

1 Diets with varying proportion of animal products: LAC <1 g/d of egg, meat, and seafood but consuming dairy products; OVO <1 g/d of meat and seafood but consuming eggs; PES <1 g/d of meat but consuming fish; LME total meat intake <50 g/d; MME: total meat intake 50–100 g/d; HME: total meat intake >100 g/d

2 Quintiles of Eat Lancet Diet Index (ELD-I); Q1 ≤4.35; Q2 4.35–21.46; Q3 21.46–37.67; Q4 37.67–59.74; Q5 >59.74

3 Quartiles of total dairy product consumption (milk, cheese, yogurt, dairy desserts): Q1: 0–96 g/d; Q2: 97–172 g/d; Q3: 173–273 g/d; Q4: 274–1429 g/d

4 Sex-specific weighted quintiles of PNNS-GS1

5 Sex-specific weighted quintiles of PNNS-GS2

6 Weighted quintiles of proportion of organic food consumption in the diet: Q1 0% organic; Q2 4% organic; Q3 17% organic; Q4 34% organic; Q5 71% organic

7 Weighted quintiles according to diet-related GHGE (kg CO_2_eq/d); Q1 0.95–2.24; Q2 2.44–3.26; Q3 3.37–4.35; Q4 4.49–5.91; Q5 6.35–11.23

8 BMI was measured

9 Value is non-adjusted mean (95% CIs)

10 System boundary as defined by study authors

∗ unit of currency was converted to euro (€) for the purpose of comparison, originally reported by authors in Swiss Franc (CHF)

∗∗ unit of currency was converted to euro (€) for the purpose of comparison, originally reported by authors in British Pound (£)

∗∗∗ functional unit converted to daily impact for the purpose of comparison, originally reported by authors as per year.

Two studies [54,72] reported that higher quality, low impact diets were neither the cheapest nor most expensive. Kesse-Guyot et al. [54] found that the pesco-vegetarian diet was the highest quality according to the cDQI and PNNS-GS2, had the lowest environmental impacts, and cost €8.94 per day. The high-meat diet was the lowest quality, had the highest impacts and cost €8.77. When these dietary patterns were optimized (outlined previously), the monetary cost decreased as follows: pescovegetarians (€6.58), high-meat eaters (€6.98). Seconda et al. [72] found that monetary cost increased across quintiles of dietary GHGE (adjustments detailed previously). Those with the lowest GHGE (Q1) and moderate guideline adherence had the lowest dietary cost (€6.89). Those in Q5, with the lowest adherence, had the highest dietary cost (€7.68). The highest adherence was in Q3 and cost €7.20 per day.

## Discussion

This review presents a synthesis of quantitative data and attempts to build upon an existing review on diet quality scores [42], by including NCD risk factors and the dietary cost associated with adherence to *a priori* dietary patterns. There are 3 principal findings; first, higher diet quality reduced planetary pressures in most studies; however, lower impact diets are not inherently optimal. High quality diets can both reduce or increase environmental impacts. Further, identifying higher quality diets that align on reductions across multiple impact indicators may be challenging. In this review, deviations were observed with energy demand [57], GHGE [60], and water use [65,68]. The latter linked to increased plant food consumption, with other studies reporting that higher consumption of PB foods increased water use [75,76], and a review highlighting that environmental co-benefits are not universal with regards to water use and sustainable diets [40].

Second, higher diet quality can result in lower BMI or BF, although this was not observed for all, and reductions in environmental impacts did not always align. The majority of populations studied in the review were in the overweight range, however research suggests that a sustainable diet could exert a potentially protective role against overweight and obesity [77].

Third, the link between higher quality diets and increased cost remains unclear due to the small number of reporting studies. Of note, of the 3 studies that did report higher financial cost, it was for adherence to FBDG [57,71], although the proportion of organic food consumed was also high [71] and the ELD [62]. Research suggests that healthy diets [78] and sustainable diets [79,80] are less affordable, particularly in lower- to middle-income countries, and those from lower SES groups [[81], [82], [83]]. However, a modelling study observed that healthy and sustainable diets (vegan, vegetarian, flexitarian) can reduce costs compared with current dietary patterns in higher-income countries. In lower-income countries, such diets would be more expensive than current diets. However, the authors did note that with policy change and food waste reductions, cost competitiveness could be achieved [84].

Based on the evidence presented, we outline some considerations for future research. First, diet quality remains a concern, with no populations reporting maximum adherence to the respective recommendations. This highlights the public health challenge of transitioning populations toward sustainable diets when the foundations of a healthy diet remain inadequate. In addition, the baseline healthiness of the populations' diets is important when measuring level of adherence to recommendations and comparing countries.

Another consideration is the difference in dietary assessment methods used. The majority of studies used a FFQ, which had been shown to underestimate environmental impact when compared to 2 24-h recalls [52] or a 7-d weighted food diary [85]. Because the amount of misreporting is unknown, the actual levels of adherence reported in studies could be lower, subsequently impacting the estimation of dietary impacts. One study summarized that if dietary variables were misreported in proportion to the misreporting of EI, GHGE were likely underestimated by 30% [77]. It also reported that under-reporting of EI appeared to confound the inverse associations with diet quality.

Another study observed that reducing EI to meet energy needs resulted in lower GHGE by up to 10% [86]. A review summarized that favorable diets in terms of sustainability could be due to lower energy content and not modifying habitual food patterns. Further, the authors proposed encouraging frugality in high-income settings as one strategy to tackle both the obesity epidemic and environmental concerns, with no prejudice on financial affordability [87]. Although moderation in energy intake is required, it should not overshadow the importance of choosing lower impact foods. In this review, the energy intake among omnivores, ovo-lacto-vegetarians, and vegans was not significantly different; however, the omnivore diet had the greatest environmental burdens [64].

The characteristics of those adopting healthier and lower environmental impact diets should be considered. The majority of studies in this review reported higher diet quality and where applicable lower impacts, in older aged participants, generally females, and those with a higher education. Additionally, they engaged in healthy lifestyle behaviors, such as being physically active and a non-smoker. The impact of SES was unclear, with one study finding that higher SES individuals exhibited high dietary GHGE levels [56] and others reporting no difference [49,51]. Based on these findings, it would be prudent for research on sustainable diets to focus on specific population groups.

The final consideration is that the production method of the food is rarely considered when estimating dietary impacts. In LCA databases, the distinction between conventional and organic farming is rare or not accurately reflected [88]. This is important because agro-ecological production can be a good proxy of biodiversity conservation, due to avoidance of chemical pesticides [89]. However, disparities on other environmental impact benefits remain [90,91]. Additionally, in this review, greater consumption of organic food [71] and greater adherence to the 2017 French guidelines, in which consumption of organic food is promoted [57], reduced dietary pesticide exposure. This finding has important implications for future dietary guidelines that promote greater consumption of plant foods, but do not always specify organic.

### Strengths and limitations

We used 7 databases and a comprehensive search string to identify the largest number of peer-reviewed papers to present the totality of the evidence. All studies reported on actual dietary patterns with the exception of one study, which also optimized the observed diets. This is important as it demonstrates that dietary change that benefits population and planetary health can occur in a culturally acceptable way. Although acceptability is one of the dimensions of a sustainable diet, it is not often emphasized in the research. Review studies [[87], [100]] highlighted that the basis for the current dialogue on sustainable diets is largely based on hypothetical dietary scenarios, which make simplistic assumptions about dietary substitutions, and lack the necessary contextualization. This was observed in the optimization study, where certain food groups were completely eliminated or significantly reduced, coupled with large increases in food groups not characteristic of usual diets [54].

There are a number of limitations to acknowledge. First, every effort was made to include all relevant literature; however, given the cross-disciplinary nature of this topic, some articles may have been omitted inadvertently. Additionally, gray literature was excluded, and only articles in the English language and those published from the year 2000 were included. The review consisted of a small number of studies, which may be explained by the specific inclusion criteria. The studies were observational, limiting their ability to draw casual conclusions due to potential biases and confounding. Although many of the studies adjusted for confounding factors, the possibility of residual confounding remains. Another concern is the representative nature of the data. All studies were conducted in higher-income countries, mostly in Europe. However, these countries are where dietary change must primarily occur to ameliorate food production impacts, and where the burden of diet-related disease is most common [31].

Another limitation is the diet quality metrics. Their conceptual differences, such as the number of components and cut-offs for scoring, may explain the heterogeneous findings. Review studies outlined how dietary scores based on binary scoring led to little consideration of the variability in food consumption, eg, MD scores. Additionally, some scores are based on a population’s median or quintile-based intakes, eg, DASH, and others based on amounts per 1000 kcal, eg, HEI [92,93]. However, examining diet quality rather than individual foods or nutrients’ contributions to health, allows interrelationships between foods and nutrients to be explored within complex dietary patterns [94]. Also, this review was focused on food-based diet quality scores, and the inclusion of nutrient-based scores may have changed findings, as some found no clear association between these scores and GHGE [95].

Other limitations include the small number of studies that calculated the cost of adherence to dietary patterns, despite being an important dimension of a sustainable diet [36]. Only one study used somewhat recent price data, and food prices are highly influenced by global externalities, which can compromise food security. Some of the BMI values were based on self-reported data, which is subject to measurement error or individual bias [96]. Studies used different dietary assessment methods, which may limit comparability [102]. The different databases, functional units, and even terminology used in relation to LCA makes comparison of studies difficult. The LCA system boundaries captured in this review were determined by the included studies, and not all aspects of the life cycle, eg, cooking, packaging, and in some cases, transport, were considered. However, production is one of the major drivers of environmental pressures within the food system [[3], [5]]. Also a large degree of uncertainty is acknowledged for all environmental impact data.

Finally, most studies assessed dietary environmental impact using 2 indicators, or in some cases a single indicator, which is not a thorough representation of a sustainable diet. Focus on certain indicators such as eutrophication, acidification, particulate matter or toxicity in the literature remains scant. The “sustainability” performance of dietary patterns is dependent on the choice of indicators selected by researchers, which should be considered when interpreting the results. It is important that future dietary patterns do not transfer the environmental burden to other resources or sectors [97]. Recent reviews have highlighted that diet and health related metrics with select climate outcomes dominate the literature. Integration of broader indicators, and linking with social and economic considerations is necessary [98,99].

## Conclusion

Unsustainable food production is a key determinant of climate change and environmental degradation. Unhealthy diets underpinned by an unsustainable food system are a contributor to the burden of disease. The prevalence of overweight and obesity remains a concern globally from both a population and planetary health perspective. As a potentially more achievable change, public health strategies should dissuade overconsumption in higher-income countries to confront the Global Syndemic. A more complex change will be shifting current population dietary patterns to a whole food, PB diet, especially when achieving a healthy diet remains challenging for many. Further, incongruities between population and planetary health can occur. Efforts on the consumption-side must be accompanied by broader changes in the food system and policies that support the production and distribution of sustainable foods.

Although research on sustainable diets is expanding rapidly, it continues to operate in silos, focusing on certain pairings, or with a discipline-specific lens, each with a hierarchy of priorities rather than examining sustainable diets in their totality. Future research is required to identify the culturally-appropriate dietary patterns that support nutritional optimization and environmental sustainability. To do this, the LCA methodology requires reporting standardization to improve robustness and comparability, as well as accounting for context-specific production practices. It is also important that a uniform set of impact indictors are integrated into research to curtail environmental burdens transferring. Equally important are greater cross-disciplinary collaborations to harmonize this research with the sociocultural and economic dimensions of sustainable diets. The affordability of future dietary patterns must be a priority for policy makers, to prevent exacerbating health inequalities.

## Acknowledgments

We thank Ms Sarah Campion for her contribution to the early stages of this review.

### Author contributions

The authors’ responsibilities were as follows – CL, SMcC, JH: conceptualized the review; CL, UL: screened the titles and abstracts, and full-texts; CL: completed the data extraction, synthesis, and quality assessment; CL: wrote and finalized the manuscript; UL, SMcC, JH: provided input on the manuscript; and all authors: read and approved the final manuscript.

This review is part of a body of papers from the SuHeGuide research project.

### Conflict of interest

SMcC and JH report no conflicts of interest.

### Funding

CL and UL report financial support under the SuHeGuide project. This project is funded through the Department of Agriculture, Food, and the Marine (DAFM)/Food Institutional Research Measure (FIRM), Ireland (2019R546), and the Department of Agriculture, Environment, and Rural Affairs (DAERA), Northern Ireland (19/R/546). DAFM and DAERA had no role in the design, analysis, or writing of this manuscript.

### Data availability

Data will be made available upon request to the corresponding author.

## Appendix A Supplementary data

Multimedia component1 Multimedia component1

Multimedia component2 Multimedia component2

## References

[1] Caron P., Ferrero Y de Loma-Osorio G., Nabarro D., Hainzelin E., Guillou M., Andersen I. (2018). Food systems for sustainable development: proposals for a profound four-part transformation. Agron. Sustain. Dev..

[2] (2019). World Population Prospects 2019 [Internet].

[3] Crippa M., Solazzo E., Guizzardi D., Monforti-Ferrario F., Tubiello F.N., Leip A. (2021). Food systems are responsible for a third of global anthropogenic GHG emissions. Nat. Food.

[4] Clark M.A., Domingo N.G.G., Colgan K., Thakrar S.K., Tilman D., Lynch J. (2020). Global food system emissions could preclude achieving the 1.5° and 2°C climate change targets. Science.

[5] Poore J., Nemecek T. (2018). Reducing food’s environmental impacts through producers and consumers. Science.

[6] Garnett T., Appleby M.C., Balmford A., Bateman I.J., Benton T.G., Bloomer P. (2013). Sustainable intensification in agriculture: premises and policies. Science.

[7] Vermeulen S.J., Campbell B.M., Ingram J.S.I. (2012). Climate change and food systems. Annu. Rev. Environ. Resour..

[8] (2019). Climate change and land: an IPCC special report on climate change, desertification, land degradation, sustainable land management, food security, and greenhouse gas fluxes in terrestrial ecosystems.

[9] (2017). Water for sustainable food and agriculture: a report produced for the G20 presidency of Germany.

[10] Damerau K., Patt A.G., van Vliet O.P.R. (2016). Water saving potentials and possible trade-offs for future food and energy supply. Glob. Environ. Change.

[11] Whitmee S., Haines A., Beyrer C., Boltz F., Capon A.G., de Souza Dias B.F. (2015). Safeguarding human health in the Anthropocene epoch: report of The Rockefeller Foundation–Lancet Commission on planetary health. Lancet.

[12] (2011). Energy-Smart” food for people and climate - issue paper.

[13] Ellis E.C., Klein Goldewijk K., Siebert S., Lightman D., Ramankutty N. (2010). Anthropogenic transformation of the biomes, 1700 to 2000. Glob. Ecol. Biogeogr..

[14] Carpenter S.R. (2005). Eutrophication of aquatic ecosystems: bistability and soil phosphorus. Proc. Natl. Acad. Sci. U. S. A..

[15] Bouwman A.F., Van Vuuren D.P., Derwent R.G., Posch M. (2002). A global analysis of acidification and eutrophication of terrestrial ecosystems. Water Air Soil Pollut.

[16] Gonzalez Fischer C., Garnett T. (2016). https://www.fao.org/documents/card/en/c/d8dfeaf1-f859-4191-954f-e8e1388cd0b7/.

[17] Goldstein B., Hansen S.F., Gjerris M., Laurent A., Birkved M. (2016). Ethical aspects of life cycle assessments of diets. Food Policy.

[18] Foley J.A., Ramankutty N., Brauman K.A., Cassidy E.S., Gerber J.S., Johnston M. (2011). Solutions for a cultivated planet. Nature.

[19] Leclère D., Obersteiner M., Barrett M., Butchart S.H.M., Chaudhary A., De Palma A. (2020). Bending the curve of terrestrial biodiversity needs an integrated strategy. Nature.

[20] Newbold T., Hudson L.N., Arnell A.P., Contu S., De Palma A., Ferrier S. (2016). Has land use pushed terrestrial biodiversity beyond the planetary boundary?. A global assessment, Science.

[21] Campbell B.M., Beare D.J., Bennett E.M., Hall-Spencer J.M., Ingram J.S.I., Jaramillo F. (2017). Agriculture production as a major driver of the Earth system exceeding planetary boundaries. Ecol. Soc..

[22] Houghton R.A. (2012). Carbon emissions and the drivers of deforestation and forest degradation in the tropics. Curr. Opin. Environ. Sustain..

[23] (2020). The state of world fisheries and aquaculture 2020. Sustainability in action.

[24] Madzorera I., Jaacks L., Paarlberg R., Herforth A., Bromage S., Ghosh S. (2021). Food systems as drivers of optimal nutrition and health: complexities and opportunities for research and implementation. Curr. Dev. Nutr..

[25] (2022). 2022 Global Nutrition Report: stronger commitments for greater action.

[26] (2019). GBD 2017 Diet Collaborators, Health effects of dietary risks in 195 countries, 1990–2017: a systematic analysis for the Global Burden of Disease Study 2017. Lancet.

[27] Willett W., Rockström J., Loken B., Springmann M., Lang T., Vermeulen S. (2019). Food in the Anthropocene: the EAT–Lancet Commission on healthy diets from sustainable food systems. Lancet.

[28] (2020). GBD 2017 Diet Collaborators, Global burden of 87 risk factors in 204 countries and territories, 1990–2019: a systematic analysis for the Global Burden of Disease Study 2019. Lancet.

[29] Swinburn B.A., Kraak V.I., Allender S., Atkins V.J., Baker P.I., Bogard J.R. (2019). The Global syndemic of obesity, undernutrition, and climate change: the Lancet Commission report. Lancet.

[30] Béné C., Oosterveer P., Lamotte L., Brouwer I.D., de Haan S., Prager S.D. (2019). When food systems meet sustainability – current narratives and implications for actions. World Dev.

[31] Springmann M., Godfray H.C.J., Rayner M., Scarborough P. (2016). Analysis and valuation of the health and climate change cobenefits of dietary change. Proc. Natl. Acad. Sci. U. S. A..

[32] Tilman D., Clark M. (2014). Global diets link environmental sustainability and human health. Nature.

[33] Kennedy E., Webb P., Block S., Griffin T., Mozaffarian D., Kyte R. (2021). Transforming food systems: the missing pieces needed to make them work. Curr. Dev. Nutr..

[34] (2019). Transforming food and agriculture to achieve the SDGs: 20 interconnected actions to guide decision-makers.

[35] Panagiotakos D. (2008). α-priori versus α-posterior methods in dietary pattern analysis: a review in nutrition epidemiology. Nutr. Bull..

[36] Monsivais P., Scarborough P., Lloyd T., Mizdrak A., Luben R., Mulligan A.A. (2015). Greater accordance with the Dietary Approaches to Stop Hypertension dietary pattern is associated with lower diet-related greenhouse gas production but higher dietary costs in the United Kingdom. Am. J. Clin. Nutr..

[37] Grosso G., Fresán U., Bes-Rastrollo M., Marventano S., Galvano F. (2020). Environmental impact of dietary choices: role of the Mediterranean and other dietary patterns in an Italian cohort. Int. J. Environ. Res. Public Health.

[38] Herforth A., Bai Y., Venkat A., Mahrt K., Ebel A., Masters W.A. (2020). https://www.fao.org/3/cb2431en/cb2431en.pdf.

[39] Page M.J., McKenzie J.E., Bossuyt P.M., Boutron I., Hoffmann T.C., Mulrow C.D. (2021). The PRISMA 2020 statement: an updated guideline for reporting systematic reviews. Int. J. Surg..

[40] Jarmul S., Dangour A.D., Green R., Liew Z., Haines A., Scheelbeek P.F. (2020). Climate change mitigation through dietary change: a systematic review of empirical and modelling studies on the environmental footprints and health effects of ‘sustainable diets’. Environ. Res. Lett..

[41] Reinhardt S.L., Boehm R., Blackstone N.T., El-Abbadi N.H., McNally Brandow J.S., Taylor S.F. (2020). Systematic review of dietary patterns and sustainability in the United States. Adv. Nutr..

[42] Hallström E., Davis J., Woodhouse A., Sonesson U. (2018). Using dietary quality scores to assess sustainability of food products and human diets: a systematic review. Ecol. Indic..

[43] Aleksandrowicz L., Green R., Joy E.J., Smith P., Haines A. (2016). The impacts of dietary change on greenhouse gas emissions, land use, water use, and health: a systematic review. PLOS ONE.

[44] Zotero Your personal research assistant. https://www.zotero.org/.

[45] Ouzzani M., Hammady H., Fedorowicz Z., Elmagarmid A. (2016). Rayyan-a web and mobile app for systematic reviews. Syst. Rev..

[46] Downes M.J., Brennan M.L., Williams H.C., Dean R.S. (2016). Development of a critical appraisal tool to assess the quality of cross-sectional studies (AXIS). BMJ Open.

[47] Study quality assessment tools (2014). https://www.nhlbi.nih.gov/health-topics/study-quality-assessment-tools.

[48] Page M.J., McKenzie J.E., Bossuyt P.M., Boutron I., Hoffmann T.C., Mulrow C.D. (2021). The PRISMA 2020 statement: an updated guideline for reporting systematic reviews. BMJ.

[49] Ridoutt B., Baird D., Hendrie G.A. (2022). Diets with higher vegetable intake and lower environmental impact: evidence from a large Australian population health survey. Nutrients.

[50] van Bussel L.M., Kuijsten A., Mars M., Feskens E.J.M., van’t Veer P. (2019). Taste profiles of diets high and low in environmental sustainability and health. Food Qual. Prefer..

[51] Ridoutt B.G., Baird D., Hendrie G.A. (2021). The role of dairy foods in lower greenhouse gas emission and higher diet quality dietary patterns. Eur. J. Nutr..

[52] Mertens E., Kuijsten A., Geleijnse J.M., Boshuizen H.C., Feskens E.J.M., van’t Veer P. (2019). FFQ versus repeated 24-h recalls for estimating diet-related environmental impact. Nutr. J..

[53] Hobbs D.A., Durrant C., Elliott J., Givens D.I., Lovegrove J.A. (2020). Diets containing the highest levels of dairy products are associated with greater eutrophication potential but higher nutrient intakes and lower financial cost in the United Kingdom. Eur. J. Nutr..

[54] Kesse-Guyot E., Allès B., Brunin J., Fouillet H., Dussiot A., Mariotti F. (2022). Nutritionally adequate and environmentally respectful diets are possible for different diet groups: an optimized study from the NutriNet-Santé cohort. Am. J. Clin. Nutr..

[55] Marty L., Chambaron S., de Lauzon-Guillain B., Nicklaus S. (2022). The motivational roots of sustainable diets: analysis of food choice motives associated to health, environmental and socio-cultural aspects of diet sustainability in a sample of French adults. Clean. Respons. Consum..

[56] Telleria-Aramburu N., Bermúdez-Marín N., Rocandio A.M., Telletxea S., Basabe N., Rebato E. (2022). Nutritional quality and carbon footprint of university students’ diets: results from the EHU12/24 study. Public Health Nutr.

[57] Kesse-Guyot E., Chaltiel D., Wang J., Pointereau P., Langevin B., Allès B. (2020). Sustainability analysis of French dietary guidelines using multiple criteria. Nat. Sustain..

[58] Perraud E., Wang J., Salomé M., Mariotti F., Kesse-Guyot E. (2023). Dietary protein consumption profiles show contrasting impacts on environmental and health indicators. Sci. Total Environ..

[59] Murakami K., Livingstone M.B.E. (2018). Greenhouse gas emissions of self-selected diets in the UK and their association with diet quality: is energy under-reporting a problem?. Nutr. J..

[60] Biesbroek S., Verschuren W.M.M., Boer J.M.A., van de Kamp M.E., van der Schouw Y.T., Geelen A. (2017). Does a better adherence to dietary guidelines reduce mortality risk and environmental impact in the Dutch sub-cohort of the European Prospective Investigation into Cancer and Nutrition?. Br. J. Nutr..

[61] Mattila T., Helin T., Antikainen R., Soimakallio S., Pingoud K., Wessman H. (2011).

[62] Kesse-Guyot E., Rebouillat P., Brunin J., Langevin B., Allès B., Touvier M. (2021). Environmental and nutritional analysis of the EAT-Lancet diet at the individual level: insights from the NutriNet-Santé study. J. Clean. Prod..

[63] Kramer G.F., Tyszler M., van’t Veer P., Blonk H. (2017). Decreasing the overall environmental impact of the Dutch diet: how to find healthy and sustainable diets with limited changes. Public Health Nutr.

[64] Rosi A., Mena P., Pellegrini N., Turroni S., Neviani E., Ferrocino I. (2017). Environmental impact of omnivorous, ovo-lacto-vegetarian, and vegan diet. Sci. Rep..

[65] Heerschop S.N., Biesbroek S., Temme E.H.M., Ocké M.C. (2021). Can healthy and sustainable dietary patterns that fit within current Dutch food habits be identified?. Nutrients.

[66] van Bussel L.M., van Rossum C.T., Temme E.H., Boon P.E., Ocké M.C. (2020). Educational differences in healthy, environmentally sustainable and safe food consumption among adults in the Netherlands. Public Health Nutr.

[67] Biesbroek S., Verschuren W.M., Boer J.M., van der Schouw Y.T., Sluijs I., Temme E.H. (2019). Are our diets getting healthier and more sustainable? Insights from the European Prospective Investigation into Cancer and Nutrition – Netherlands (EPIC-NL) cohort. Public Health Nutr.

[68] Vellinga R.E., van de Kamp M., Toxopeus I.B., van Rossum C.T.M., de Valk E., Biesbroek S. (2019). Greenhouse gas emissions and blue water use of Dutch diets and its association with health. Sustainability.

[69] Biesbroek S., Monique Verschuren W.M., van der Schouw Y.T., Sluijs I., Boer J.M.A., Temme E.H.M. (2018). Identification of data-driven Dutch dietary patterns that benefit the environment and are healthy. Clim. Change.

[70] Marty L., de Lauzon-Guillain B., Nicklaus S. (2022). Short- and mid-term impacts of COVID-19 outbreak on the nutritional quality and environmental impact of diet. Front. Nutr..

[71] Baudry J., Pointereau P., Seconda L., Vidal R., Taupier-Letage B., Langevin B. (2019). Improvement of diet sustainability with increased level of organic food in the diet: findings from the BioNutriNet cohort. Am. J. Clin. Nutr..

[72] Seconda L., Baudry J., Allès B., Boizot-Szantai C., Soler L.G., Galan P. (2018). Comparing nutritional, economic, and environmental performances of diets according to their levels of greenhouse gas emissions. Clim. Change.

[73] Frehner A., Zanten H.H.E.V., Schader C., Boer I.J.M.D., Pestoni G., Rohrmann S. (2021). How food choices link sociodemographic and lifestyle factors with sustainability impacts. J. Clean. Prod..

[74] Laine J.E., Huybrechts I., Gunter M.J., Ferrari P., Weiderpass E., Tsilidis K. (2021). Co-benefits from sustainable dietary shifts for population and environmental health: an assessment from a large European cohort study. Lancet Planet. Health.

[75] Meier T., Christen O. (2013). Environmental impacts of dietary recommendations and dietary styles: Germany as an example. Environ. Sci. Technol..

[76] Springmann M., Wiebe K., Mason-D’Croz D., Sulser T.B., Rayner M., Scarborough P. (2018). Health and nutritional aspects of sustainable diet strategies and their association with environmental impacts: a global modelling analysis with country-level detail. Lancet Planet. Health.

[77] Seconda L., Egnell M., Julia C., Touvier M., Hercberg S., Pointereau P. (2020). Association between sustainable dietary patterns and body weight, overweight, and obesity risk in the NutriNet-Santé prospective cohort. Am. J. Clin. Nutr..

[78] Rao M., Afshin A., Singh G., Mozaffarian D. (2013). Do healthier foods and diet patterns cost more than less healthy options? A systematic review and meta-analysis. BMJ Open.

[79] Drewnowski A. (2020). Analysing the affordability of the EAT–Lancet diet. Lancet Glob. Health.

[80] Hirvonen K., Bai Y., Headey D., Masters W.A. (2020). Affordability of the EAT–Lancet reference diet: a global analysis. Lancet Glob Health.

[81] Gupta S., Vemireddy V., Singh D.K., Pingali P. (2021). Ground truthing the cost of achieving the EAT lancet recommended diets: evidence from rural India. Glob. Food Sec..

[82] Darmon N., Drewnowski A. (2015). Contribution of food prices and diet cost to socioeconomic disparities in diet quality and health: a systematic review and analysis. Nutr. Rev..

[83] Barosh L., Friel S., Engelhardt K., Chan L. (2014). The cost of a healthy and sustainable diet–who can afford it?. Aust. N. Z. J. Public Health.

[84] Springmann M., Clark M.A., Rayner M., Scarborough P., Webb P. (2021). The global and regional costs of healthy and sustainable dietary patterns: a modelling study. Lancet Planet. Health.

[85] Sjörs C., Raposo S.E., Sjölander A., Bälter O., Hedenus F., Bälter K. (2016). Diet-related greenhouse gas emissions assessed by a food frequency questionnaire and validated using 7-day weighed food records. Environ. Health.

[86] Vieux F., Darmon N., Touazi D., Soler L.G. (2012). Greenhouse gas emissions of self-selected individual diets in France: changing the diet structure or consuming less?. Ecol. Econ..

[87] Perignon M., Vieux F., Soler L.G., Masset G., Darmon N. (2017). Improving diet sustainability through evolution of food choices: review of epidemiological studies on the environmental impact of diets. Nutr. Rev..

[88] Montemayor E., Andrade E.P., Bonmatí A., Antón A. (2022). Critical analysis of life cycle inventory datasets for organic crop production systems. Int. J. Life Cycle Assess..

[89] Gong S., Hodgson J.A., Tscharntke T., Liu Y., van der Werf W., Batáry P. (2022). Biodiversity and yield trade-offs for organic farming. Ecol. Lett..

[90] Clark M., Tilman D. (2017). Comparative analysis of environmental impacts of agricultural production systems, agricultural input efficiency, and food choice. Environ. Res. Lett..

[91] Muller A., Schader C., El-Hage Scialabba N., Brüggemann J., Isensee A., Erb K.H. (2017). Strategies for feeding the world more sustainably with organic agriculture. Nat. Commun..

[92] Burggraf C., Teuber R., Brosig S., Meier T. (2018). Review of a priori dietary quality indices in relation to their construction criteria. Nutr. Rev..

[93] Waijers P.M.C.M., Feskens E.J.M., Ocké M.C. (2007). A critical review of predefined diet quality scores. Br. J. Nutr..

[94] Hu F.B. (2002). Dietary pattern analysis: a new direction in nutritional epidemiology. Curr. Opin. Lipidol..

[95] Payne C.L., Scarborough P., Cobiac L. (2016). Do low-carbon-emission diets lead to higher nutritional quality and positive health outcomes? A systematic review of the literature. Public Health Nutr.

[96] Stommel M., Schoenborn C.A. (2009). Accuracy and usefulness of BMI measures based on self-reported weight and height: findings from the NHANES and NHIS 2001-2006. BMC Public Health.

[97] Aldaya M.M., Ibañez F.C., Domínguez-Lacueva P., Murillo-Arbizu M.T., Rubio-Varas M., Soret B. (2021). Indicators and recommendations for assessing sustainable healthy diets. Foods.

[98] Webb P., Livingston Staffier K., Lee H., Howell B., Battaglia K., Bell B.M. (2023). Measurement of diets that are healthy, environmentally sustainable, affordable, and equitable: a scoping review of metrics, findings, and research gaps. Front. Nutr..

[99] Machado P., McNaughton S.A., Livingstone K.M., Hadjikakou M., Russell C., Wingrove K. (2023). Measuring adherence to sustainable healthy diets: a scoping review of dietary metrics. Adv. Nutr..

[100] Ridoutt B.G., Hendrie G.A., Noakes M. (2017). Dietary strategies to reduce environmental impact: a critical review of the evidence base. Adv. Nutr..

[101] Bray G., Bouchard C., James W.P.T., Bray G., Bouchard C., James W.P.T. (1998). Handbook of Obesity.

[102] Bailey R.L. (2021). Overview of dietary assessment methods for measuring intakes of foods, beverages, and dietary supplements in research studies. Curr. Opin. Biotechnol..

