# Supplementary material for: Aligning Environmental Sustainability, Health Outcomes, and Affordability in Diet Quality: A Systematic Review
Source: Adv Nutr. 2023 Jul 31;14(6):1270–96. doi: 10.1016/j.advnut.2023.07.007 (PMC10721486; doi:10.1016/j.advnut.2023.07.007)
Supplement: Multimedia component1 [file mmc1.docx]

**Aligning Environmental Sustainability, Health Outcomes and Affordability in Diet Quality: A Systematic Review**

Clarissa L Leydon, Ursula M Leonard, Sinéad N McCarthy, and Janas M Harrington

***Online Supplementary Material***

**Table of Contents**

[**PRISMA 2020 Abstract Checklist** 2](#_Toc144136133)

[**PRISMA 2020 Checklist** 3](#_Toc144136134)

[**Table S1 Database Search Terms** 7](#_Toc144136135)

[**Description of Diet Quality Metrics** 11](#_Toc144136136)

[**Table S2 AXIS Critical Appraisal of Cross-Sectional Studies** 17](#_Toc144136137)

[**Table S3 NIH Quality Assessment Tool for Observational Cohort and Cross-sectional Studies** 19](#_Toc144136138)

[**References** 20](#_Toc144136139)

# **PRISMA 2020 Abstract Checklist**

| **Section and Topic** | **Item #** | **Checklist item** | **Reported (Yes/No)** |
| --- | --- | --- | --- |
| **TITLE** | | |  |
| Title | 1 | Identify the report as a systematic review. | Yes |
| **BACKGROUND** | | |  |
| Objectives | 2 | Provide an explicit statement of the main objective(s) or question(s) the review addresses. | Yes |
| **METHODS** | | |  |
| Eligibility criteria | 3 | Specify the inclusion and exclusion criteria for the review. | No |
| Information sources | 4 | Specify the information sources (e.g. databases, registers) used to identify studies and the date when each was last searched. | Yes |
| Risk of bias | 5 | Specify the methods used to assess risk of bias in the included studies. | Yes |
| Synthesis of results | 6 | Specify the methods used to present and synthesise results. | Yes |
| **RESULTS** | | |  |
| Included studies | 7 | Give the total number of included studies and participants and summarise relevant characteristics of studies. | Yes |
| Synthesis of results | 8 | Present results for main outcomes, preferably indicating the number of included studies and participants for each. If meta-analysis was done, report the summary estimate and confidence/credible interval. If comparing groups, indicate the direction of the effect (i.e. which group is favoured). | Yes |
| **DISCUSSION** | | |  |
| Limitations of evidence | 9 | Provide a brief summary of the limitations of the evidence included in the review (e.g. study risk of bias, inconsistency and imprecision). | No |
| Interpretation | 10 | Provide a general interpretation of the results and important implications. | Yes |
| **OTHER** | | |  |
| Funding | 11 | Specify the primary source of funding for the review. | No |
| Registration | 12 | Provide the register name and registration number. | No |

# **PRISMA 2020 Checklist**

| **Section and Topic** | **Item #** | **Checklist item** | **Location where item is reported** |
| --- | --- | --- | --- |
| **TITLE** | | |  |
| Title | 1 | Identify the report as a systematic review. | Page 1 |
| **ABSTRACT** | | |  |
| Abstract | 2 | See the PRISMA 2020 for Abstracts checklist. | Supplementary Material File 1, Page 2 |
| **INTRODUCTION** | | |  |
| Rationale | 3 | Describe the rationale for the review in the context of existing knowledge. | Pages 5-6 |
| Objectives | 4 | Provide an explicit statement of the objective(s) or question(s) the review addresses. | Page 9 |
| **METHODS** | | |  |
| Eligibility criteria | 5 | Specify the inclusion and exclusion criteria for the review and how studies were grouped for the syntheses. | Pages 7 (Table 1) & 9 |
| Information sources | 6 | Specify all databases, registers, websites, organisations, reference lists and other sources searched or consulted to identify studies. Specify the date when each source was last searched or consulted. | Page 7 |
| Search strategy | 7 | Present the full search strategies for all databases, registers and websites, including any filters and limits used. | Supplementary Material File 1, Pages 7-10 (Table S1) |
| Selection process | 8 | Specify the methods used to decide whether a study met the inclusion criteria of the review, including how many reviewers screened each record and each report retrieved, whether they worked independently, and if applicable, details of automation tools used in the process. | Pages 7 & 8 |
| Data collection process | 9 | Specify the methods used to collect data from reports, including how many reviewers collected data from each report, whether they worked independently, any processes for obtaining or confirming data from study investigators, and if applicable, details of automation tools used in the process. | Page 8 |
| Data items | 10a | List and define all outcomes for which data were sought. Specify whether all results that were compatible with each outcome domain in each study were sought (e.g. for all measures, time points, analyses), and if not, the methods used to decide which results to collect. | Page 8 & Supplementary Material File 2 |
|  | 10b | List and define all other variables for which data were sought (e.g. participant and intervention characteristics, funding sources). Describe any assumptions made about any missing or unclear information. | Supplementary Material File 2 |

| Study risk of bias assessment | 11 | | Specify the methods used to assess risk of bias in the included studies, including details of the tool(s) used, how many reviewers assessed each study and whether they worked independently, and if applicable, details of automation tools used in the process. | | Page 8 |
| --- | --- | --- | --- | --- | --- |
| Effect measures | 12 | | Specify for each outcome the effect measure(s) (e.g. risk ratio, mean difference) used in the synthesis or presentation of results. | | - |
| Synthesis methods | 13a | | Describe the processes used to decide which studies were eligible for each synthesis (e.g. tabulating the study intervention characteristics and comparing against the planned groups for each synthesis (item #5)). | | - |
|  | 13b | | Describe any methods required to prepare the data for presentation or synthesis, such as handling of missing summary statistics, or data conversions. | | - |
|  | 13c | | Describe any methods used to tabulate or visually display results of individual studies and syntheses. | | Page 9 |
|  | 13d | | Describe any methods used to synthesize results and provide a rationale for the choice(s). If meta-analysis was performed, describe the model(s), method(s) to identify the presence and extent of statistical heterogeneity, and software package(s) used. | | Page 9 |
|  | 13e | | Describe any methods used to explore possible causes of heterogeneity among study results (e.g. subgroup analysis, meta-regression). | | - |
|  | 13f | | Describe any sensitivity analyses conducted to assess robustness of the synthesized results. | | - |
| Reporting bias assessment | 14 | | Describe any methods used to assess risk of bias due to missing results in a synthesis (arising from reporting biases). | | - |
| Certainty assessment | 15 | | Describe any methods used to assess certainty (or confidence) in the body of evidence for an outcome. | | - |
| **RESULTS** | | | |  | |
| Study selection | 16a | Describe the results of the search and selection process, from the number of records identified in the search to the number of studies included in the review, ideally using a flow diagram. | | Page 10 (Figure 1) | |
|  | 16b | Cite studies that might appear to meet the inclusion criteria, but which were excluded, and explain why they were excluded. | | - | |
| Study characteristics | 17 | Cite each included study and present its characteristics. | | Table 2 | |
| Risk of bias in studies | 18 | Present assessments of risk of bias for each included study. | | Supplementary Material File 1, Pages 17-20 (Table S2 & S3) | |

| Results of individual studies | 19 | For all outcomes, present, for each study: (a) summary statistics for each group (where appropriate) and (b) an effect estimate and its precision (e.g. confidence/credible interval), ideally using structured tables or plots. | Tables 3 - 8 |
| --- | --- | --- | --- |
| Results of syntheses | 20a | For each synthesis, briefly summarise the characteristics and risk of bias among contributing studies. | - |
|  | 20b | Present results of all statistical syntheses conducted. If meta-analysis was done, present for each the summary estimate and its precision (e.g. confidence/credible interval) and measures of statistical heterogeneity. If comparing groups, describe the direction of the effect. | - |
|  | 20c | Present results of all investigations of possible causes of heterogeneity among study results. | - |
|  | 20d | Present results of all sensitivity analyses conducted to assess the robustness of the synthesized results. | - |
| Reporting biases | 21 | Present assessments of risk of bias due to missing results (arising from reporting biases) for each synthesis assessed. | - |
| Certainty of evidence | 22 | Present assessments of certainty (or confidence) in the body of evidence for each outcome assessed. | - |
| **DISCUSSION** | | |  |
| Discussion | 23a | Provide a general interpretation of the results in the context of other evidence. | Pages 27-28 |
|  | 23b | Discuss any limitations of the evidence included in the review. | Pages 30-32 |
|  | 23c | Discuss any limitations of the review processes used. | Pages 30-32 |
|  | 23d | Discuss implications of the results for practice, policy, and future research. | Pages 28-30 |
| **OTHER INFORMATION** | | |  |
| Registration and protocol | 24a | Provide registration information for the review, including register name and registration number, or state that the review was not registered. | Page 7 |
|  | 24b | Indicate where the review protocol can be accessed, or state that a protocol was not prepared. | Page 7 |
|  | 24c | Describe and explain any amendments to information provided at registration or in the protocol. | See PROSPERO <http://www.crd.york.ac.uk/PROSPERO>  (ID = CRD42021238055) |
| Support | 25 | Describe sources of financial or non-financial support for the review, and the role of the funders or sponsors in the review. | Page 33 |
| Competing interests | 26 | Declare any competing interests of review authors. | Page 33 |
| Availability of data, code and other materials | 27 | Report which of the following are publicly available and where they can be found: template data collection forms; data extracted from included studies; data used for all analyses; analytic code; any other materials used in the review. | Supplementary Material File 2 |

# **Table S1 Database Search Terms**

Detailed information on the searches performed in the databases are documented below.

| Database | Search Syntax | Filter(s) |
| --- | --- | --- |
| CINAHL Plus (EBSCO) | ("diet* quality" OR "diet* pattern*" OR "diet* score*" OR "adequacy index*" OR "diet quality index*" OR "diet* pattern score*" OR "diet quality score" OR "diet* index*" OR "diet* adherence" OR "sustainable diet*" OR "food-based diet quality score*" OR "food-based diet quality index*" OR "food-based diet quality index*" OR "diet* adherence" OR "a priori" OR "a priori dietary pattern*" OR (MH "Eating Behavior"))  AND ("environmental impact*" OR "life cycle assessment*" OR "life cycle analysis" OR (MH "Greenhouse Gases") OR (MH "Carbon Footprint") OR (MH "WaterFootprint") OR "environmental footprint*" OR (MH "Environmental Sustainability") OR "climate impact*" OR "land use" OR "water use" OR "water footprint*" OR "eutrophication" OR "acidification" OR (MH "Conservation of Natural Resources") OR (MH "Ecosystem") OR "biodivers*" OR (MH "Greenhouse Effect") OR "environmental indicator*" OR (MH "Environmental Monitoring"))  AND ((MH "Health") OR "health outcomes" OR (MH "Health Impact Assessment") OR (MH "Cardiovascular Diseases") OR (MH "Neoplasms") OR (MH "Diabetes Mellitus") OR (MH "Obesity") OR (MH "Coronary Disease") OR "all cause mortality" OR "risk* factor*" OR "metabolic risk* factor*" OR "Noncommunicable disease*" OR "Non-communicable disease*" OR (MH "Hypertension") OR (MH "Stroke"") OR (MH "Morbidity") OR (MH "Prevalence") OR (MH "Incidence") OR "risk" OR (MH "Mortality")) | Year,  Language |
| Cochrane Library | #1 MeSH descriptor: [Diet, Healthy]  #2 "diet* quality" OR "diet* pattern*" OR "diet* score*" OR "adequacy index*" OR "diet quality index*" OR "diet* pattern score*" OR "diet quality score" OR "diet* index*" OR "diet* adherence" OR "sustainable diet*" OR "food-based diet quality score*" OR "food-based diet quality index*" OR "food-based diet quality index*" OR "diet* adherence" OR "a priori" OR "a priori dietary pattern*"  #3 #1 OR #2  #4 MeSH descriptor: [Greenhouse Effect]  #5 MeSH descriptor: [Carbon Footprint]  #6 MeSH descriptor: [Environmental Monitoring]  #7 MeSH descriptor: [Conservation of Natural Resources]  #8 MeSH descriptor: [Biodiversity]  #9 "environmental impact*" OR "sustainab*" OR "Life cycle assessment" OR "Life cycle analysis" OR "greenhouse gas*" OR "greenhouse gas emission*" OR "carbon footprint" OR "climate impact" OR "environmental footprint*" OR "land use" OR "water use" OR "water footprint*" OR "eutrophication" OR "acidification" OR "natural resource*", OR "ecosystem" OR "biodivers*" OR "Greenhouse Effect" OR "Environmental Indicators" OR "Environmental Monitoring" OR "Conservation of Natural Resources"  #10 #4 OR #5 OR #6 OR #7 OR #8 OR #9  #11 MeSH descriptor: [Health]  #12 MeSH descriptor: [Health Impact Assessment]  #13 MeSH descriptor: [Cardiovascular Diseases]  #14 MeSH descriptor: [Coronary Disease]  #15 MeSH descriptor: [Feeding Behavior]  #16 MeSH descriptor: [Diabetes Mellitus]  #17 MeSH descriptor: [Obesity]  #18 MeSH descriptor: [Risk Factors]  #19 MeSH descriptor: [Noncommunicable Diseases]  #20 MeSH descriptor: [Hypertension]  #21 MeSH descriptor: [Stroke]  #22 MeSH descriptor: [Risk]  #23 MeSH descriptor: [Mortality]  #24 MeSH descriptor: [Morbidity]  #25 "health" OR "health outcome*" OR "health impact" OR "cardiovascular disease" OR "cancer" OR "diabetes mellitus" OR "diabetes", "overweight" OR "obesity" OR "coronary heart disease" OR "all-cause mortality", OR "hypertension", OR "stroke" OR "prevalence" OR "incidence" OR "risk" OR "rate" OR "mortality" OR "morbidity" OR "risk factor*" OR "metabolic risk* factor*" OR "Noncommunicable disease*" OR "Non-communicable disease*"  #26 #11 OR #12 OR #13 OR #14 OR #15 OR #16 OR #17 OR #18 OR #19 OR #20 OR #21 OR #22 OR #23 OR #24 OR #25 1100072  #27 #3 AND #10 AND #26 | Year |
| Embase | ('diet* quality' OR 'dietary pattern'/exp OR 'dietary pattern' OR 'diet* score' OR 'adequacy index*' OR 'diet quality index'/exp OR 'diet quality index' OR 'diet* pattern score*' OR 'diet quality score'/exp OR 'diet quality score' OR 'diet* index*' OR 'dietary compliance'/exp OR 'dietary compliance' OR 'sustainable diet*' OR 'food-based diet quality score*' OR 'food-based diet quality index*' OR 'food-based diet quality index*'OR "diet* adherence" OR "a priori" OR "a priori dietary pattern*" )  AND ('environmental impact'/exp OR 'environmental impact' OR 'environmental sustainability'/exp OR 'environmental sustainability' OR 'life cycle assessment'/exp OR 'life cycle assessment' OR 'life cycle'/exp OR 'life cycle' OR 'greenhouse gas'/exp OR 'greenhouse gas' OR 'greenhouse gas emission*' OR 'carbon footprint'/exp OR 'carbon footprint' OR 'environmental footprint'/exp OR 'environmental footprint' OR 'climate impact' OR 'land use'/exp OR 'land use' OR 'water footprint'/exp OR 'water footprint' OR 'eutrophication'/exp OR eutrophication OR 'acidification'/exp OR acidification OR 'environmental protection'/exp OR 'environmental protection' OR 'ecosystem'/exp OR ecosystem OR 'biodiversity'/exp OR biodiversity OR 'greenhouse effect'/exp OR 'greenhouse effect' OR 'environmental indicator'/exp OR 'environmental indicator' OR 'environmental monitoring'/exp OR 'environmental monitoring')  AND ('health'/exp OR health OR 'health outcomes'/exp OR 'health outcomes' OR 'health impact assessment'/exp OR 'health impact assessment' OR 'cardiovascular disease'/exp OR 'cardiovascular disease' OR 'malignant neoplasm'/exp OR 'malignant neoplasm' OR 'diabetes mellitus'/exp OR 'diabetes mellitus' OR 'obesity'/exp OR obesity OR 'ischemic heart disease'/exp OR 'ischemic heart disease' OR 'all cause mortality'/exp OR 'all cause mortality' OR 'hypertension'/exp OR hypertension OR 'cerebrovascular accident'/exp OR 'cerebrovascular accident' OR 'incidence'/exp OR incidence OR 'prevalence'/exp OR prevalence OR 'risk'/exp OR risk OR 'mortality'/exp OR mortality OR 'morbidity'/exp OR morbidity OR 'risk factor'/exp OR 'risk factor' OR "metabolic risk* factor*" OR 'non communicable disease'/exp) | Year,  Language |
| Greenfile (EBSCO) | ("diet* quality" OR "diet* pattern*" OR "diet* score*" OR "adequacy index*" OR "diet quality index*" OR "diet* pattern score*" OR "diet quality score" OR "diet* index*" OR "diet* adherence" OR "sustainable diet*" OR 'food-based diet quality score*' OR 'food-based diet quality index*' OR 'food-based diet quality index*'OR "diet* adherence" OR "a priori" OR "a priori dietary pattern*" )  AND ( "environmental impact*" OR "sustainab*" OR "Life cycle assessment" OR "Life cycle analysis" OR "greenhouse gas*" OR "greenhouse gas emission*" OR "carbon footprint" OR "environmental footprint*"OR "climate impact*" OR "land use" OR "water use" OR 'water footprint*' OR eutrophication OR acidification OR natural resource*, OR ecosystem OR biodivers* OR "Greenhouse Effect" OR "Environmental Indicators" OR "Environmental Monitoring" OR "Conservation of Natural Resources" )  AND ("health" OR "health outcome*" OR "health impact" OR "cardiovascular disease" OR "cancer" OR "diabetes mellitus" OR "diabetes", "overweight" OR "obesity" OR "coronary heart disease" OR "all-cause mortality", OR "risk* factor*" OR "metabolic risk* factor*" OR "Noncommunicable disease*" OR "Non-communicable disease*" OR "hypertension", OR "stroke" OR "prevalence" OR "incidence" OR "risk" OR "rate" OR "mortality" OR "morbidity" ) | Year,  Language |
| PubMed | ("diet* quality" OR "diet* pattern*" OR "diet* score*" OR "adequacy index*" OR "diet quality index*" OR "food-based diet quality score*" OR "food-based diet quality index*" OR "diet* pattern score*" OR "diet quality score" OR "diet* index*" OR "diet* adherence" OR "sustainable diet*" OR "a priori" OR "a priori dietary pattern*" OR "Diet, Healthy"[Mesh] OR "Feeding Behavior"[Mesh])  AND ("environmental impact*" OR "sustainab*" OR "Life cycle assessment" OR "Life cycle analysis" OR "greenhouse gas*" OR "greenhouse gas emission*" OR "carbon footprint" OR "environmental footprint*"OR "climate impact*" OR "land use" OR "water use" OR 'water footprint*' OR "eutrophication" OR "acidification" OR "natural resource*", OR "ecosystem" OR "biodivers*" OR "Greenhouse Effect"[Mesh] OR "Greenhouse Gases"[Mesh] OR "Carbon Footprint"[Mesh] OR "Environmental Indicators"[Mesh] OR "Environmental Monitoring"[Mesh] OR "Conservation of Natural Resources"[Mesh])  AND ("health" OR "health outcome*" OR "health impact" OR "cardiovascular disease" OR "cancer" OR "diabetes mellitus" OR "diabetes", "overweight" OR "obesity" OR "coronary heart disease" OR "all-cause mortality", OR "hypertension", OR "stroke" OR "prevalence" OR "incidence" OR "risk" OR "rate" OR "risk* factor*" OR "metabolic risk* factor*" OR "mortality" OR "morbidity" OR "Noncommunicable disease*" OR "Non-communicable disease*" OR "cardiovascular diseases" [MeSH] OR "diabetes mellitus" [MeSH] OR "diabetes mellitus, type 2" [MeSH] OR "obesity" [Mesh]) | Year, Language, Humans |
| Scopus | TITLE-ABS-KEY ("diet* quality" OR "diet* pattern*" OR "diet* score*" OR "adequacy index*" OR "diet quality index*" OR "food-based diet quality score*" OR "food-based diet quality index*" OR "diet* pattern score*" OR "diet quality score" OR "diet* index*" OR "diet* adherence" OR "sustainable diet*" OR "a priori" OR "a priori diet* pattern*")  AND TITLE-ABS-KEY ("environmental impact*" OR "sustainab*" OR "Life cycle assessment" OR "Life cycle analysis" OR "greenhouse gas*" OR "greenhouse gas emission*" OR "carbon footprint" OR "environmental footprint*" OR "climate impact*" OR "land use" OR "water use" OR "water footprint*" OR "eutrophication" OR "acidification" OR "natural resource*" OR "ecosystem" OR "biodivers*" OR "Greenhouse Effect" OR "Environmental Indicators" OR "Environmental Monitoring" OR "Conservation of Natural Resources")  AND TITLE-ABS-KEY ("health" OR "health outcome*" OR "health impact" OR "cardiovascular disease" OR "cancer" OR "diabetes mellitus" OR "diabetes" OR "overweight" OR "obesity" OR "coronary heart disease" OR "all-cause mortality" OR "hypertension" OR "stroke" OR "prevalence" OR "incidence" OR "risk" OR "rate" OR "risk* factor*" OR "metabolic risk* factor*" OR "mortality" OR "morbidity" OR "Noncommunicable disease*" OR "Non-communicable disease*") | Year,  Language |
| Web of Science | TS=("diet* quality" OR "diet* pattern*" OR "diet* score*" OR "adequacy index*" OR "diet quality index*" OR "food-based diet quality score*" OR "food-based diet quality index*" OR "diet* pattern score*" OR "diet quality score" OR "diet* index*" OR "diet* adherence" OR "sustainable diet*" OR "a priori" OR "a priori diet* pattern*")  AND TS=("environmental impact*" OR "sustainab*" OR "Life cycle assessment" OR "Life cycle analysis" OR "greenhouse gas*" OR "greenhouse gas emission*" OR "carbon footprint" OR "environmental footprint*"OR "climate impact*" OR "land use" OR "water use" OR "water footprint*" OR eutrophication OR acidification OR natural resource*, OR ecosystem OR biodivers* OR "Greenhouse Effect" OR "Environmental Indicators" OR "Environmental Monitoring" OR "Conservation of Natural Resources")  AND TS=("health" OR "health outcome*" OR "health impact" OR "cardiovascular disease" OR "cancer" OR "diabetes mellitus" OR "diabetes", "overweight" OR "obesity" OR "coronary heart disease" OR "all-cause mortality", OR "hypertension", OR "stroke" OR "prevalence" OR "incidence" OR "risk" OR "rate" OR "risk* factor*" OR "metabolic risk* factor*" OR "mortality" OR "morbidity" OR "Noncommunicable disease*" OR "Non-communicable disease*")  TS=Topic | Year,  Language |

# **Description of Diet Quality Metrics**

Due to the variability of the diet quality metrics, they were broadly classified as follows:

-**Group A:** Based on adherence to international nutrition guidelines or national dietary guidelines

-**Group B:** Based on region specific health-improving diets

-**Group C:** Based on dietary patterns to lower risk of chronic disease

-**Group D:** Based on a universal reference diet to promote both population and planetary health

**Group A**

*Dietary Guideline Index*

The Dietary Guideline Index (DGI) developed by Golley, Hendrie and McNaughton (2014) (1) was used in two studies (2,3) and adapted to reflect the dietary intake targets in the 2013 Australian Dietary Guidelines (ADG) for Adults. The index reflects the amount and quality of food consumed from core food groups, discretionary foods and beverages, the quality of some food choices (low fat dairy, wholegrain, and type of spread), and variety of foods consumed within each core food group. The DGI is comprised of 11 components and individuals receive a diet quality score out of 100, where a higher score reflects greater conformance with the guidelines.

*Dutch Healthy Diet Index*

The Dutch Healthy Diet Index (DHD-Index) is an instrument to measure adherence to the Dutch Guidelines for a Healthy Diet, and was reported by eight studies. All studies adopted an index, which reflects the dietary guidelines of 2015 (DHD15-index). The DHD15-index consists of 15 components representing the fifteen food-based Dutch dietary guidelines of 2015. For each component, a maximum of ten points could be allotted, resulting in a total score ranging from zero to 150 points. The components vegetables, fruit, legumes, nuts, fish and tea are adequacy components, and the components red meat, processed meat, sugar sweetened beverages (SSB) and fruit juices, sodium, and alcohol are moderation components. The component dairy is an optimum component with an optimal range of intakes, whereas the fats and oils component is defined as a ratio component to reflect replacement of intake of less desired foods with healthier options in that food group. The coffee component is defined as a qualitative component based on type of coffee. The wholegrain products component is scored based on two sub-components as there are two guidelines for grain products: an adequacy component for wholegrain consumption and a ratio component to reflect replacement of refined grain products by wholegrain products (4). Seven studies used modified versions of the tool, six omitted the coffee component as the consumption data used did not distinguish type (5–10), one omitted the coffee and salt components and adapted the wholegrain, red meat, and processed meat components (11).

*Healthy Eating Index*

The Healthy Eating Index (HEI) is a measure of diet quality used to assess how well a set of food items align with key recommendations of the 2010 Dietary Guidelines for Americans (HEI-2010) and was reported by one study (12). The HEI-2010 consists of 12 components, including nine on adequacy (fruit, whole fruit, vegetables, greens and beans, wholegrains, dairy, protein foods, seafood and plant proteins, fatty acids) and three on moderation (refined grains, sodium, empty calories), that are scored per 1000 kcal or as a percentage of calories. The theoretical range of the HEI-2010 is from zero to 100 (13).

*Programme National Nutrition Santé Guideline Score*

Seven studies reported adherence to the French national recommendations of the Programme National Nutrition Santé. Three studies used a modified PNNS Guideline Score (mPNNS-GS1) based on the 2001 food-based dietary guidelines (FBDGs) (14), with the removal of the physical activity component (15–17). The mPNNS-GS1 includes 12 components: eight refer to adequacy recommendations (fruit and vegetables, starchy foods, whole-grain products, dairy products, meat and poultry and eggs, seafood, vegetable fat, and water vs. SSB) and four to moderation (added fat, salt, sweets, and alcohol) (14). The theoretical range is -17 to 13.5 points. The updated PNNS-GS2 metric, which is based on the 2017 FBDGs, was used in five studies (15,18–21). The PNNS-GS2 consists of 13 components: six of adequacy recommendations (fruit and vegetables, nuts, legumes, whole-grain products, dairy products, and fish and seafood) and seven of moderation (red meat, processed meat, sugary products, SSB, added fat, salt, and alcoholic beverages) (22). Physical activity is not part of the score. The theoretical range is -17 to 13.5 points. For both PNNS-GS1 and PNNS-GS2, if reported energy intake was >5% or more of the calculated energy need, the total score was reduced by the same proportion. Given the penalisation on energy intake, the theoretical range of both scores may be lower than the threshold (22). One study used the original PNNS-GS2 (15), one used the simplified version (sPNNS-GS2) (range -17 to 13.5) (19), which includes only the principal recommendations (i.e., excluding components related to organic food, fatty fish, and recommended oils ratio). Two studies modified the PNNS-GS2 (15,18), with a range of -17 to 14.25. These studies included a linear allocation of half-points to improve discrimination power between cut-offs. An exception concerned milk and dairy products, and fish for which the relationship to global health has been found non-linear, hence a parabolic-shaped relationship in allocated points (15). Two used a modified version of sPNNS-GS2 (range -17 to 11.5), which excluded bonus points for added fat below 16% of energy intake (20,21).

**Group B**

*Mediterranean Diet*

Four studies reported compliance to the Mediterranean diet (MD) each using a different metric; the Literature-Based Adherence Score to the Mediterranean Diet (LAMD) (19), a large-scale score on inherent characteristics (12), a modified version of the original MD score (23), and an Italian Mediterranean Diet (IMD) Index (24). The LAMD is based on an updated systematic review and meta-analysis of cohort studies investigating adherence to the Mediterranean diet and health status. Data obtained from the studies provided the cut-off value for consumption of each food group in order to propose an estimation of adherence to the MD, based on descriptive data of the literature. The LAMD score includes nine food categories; three negative components (meat, dairy products, and alcohol) and six positive components (fruits, vegetables, legumes, cereals, fish, and olive oil). With a score ranging from zero (lowest adherence) to 18 (highest adherence) (25). The large-scale MD score is based on the weekly consumption of 11 food groups; seven related to items that characterise the MD (non-refined cereals, fruits, vegetables, legumes, potatoes, fish and olive oil), three related to items which diverge from this diet pattern (meat and meat-products, poultry and full-fat dairy products), and moderate alcohol consumption. The food items are scored according to the recommended frequency of intake defined by the MD pyramid. The total score ranged from zero to 55 (26).

Trichopoulou et al. (27) developed a modified score based on the original MD score, by the same authors. This modified score allows application to non-Mediterranean populations, in which intake of monounsaturated fats from olive oil is minimal. The score substituted monounsaturated lipids with the sum of monounsaturated and polyunsaturated lipids in the numerator of the lipid ratio. The sum of monounsaturated and polyunsaturated fats to saturated fats was calculated. It consists of nine components; six protective components (fatty acid ratio, legumes, grains, fruits, nuts and seeds, vegetables, and fish), two non-protective components (dairy products or meat) and alcohol. Participants were classified for each component into sex-specific quintiles by intake. For dairy products and meat, a score of one was assigned to intake below or equal to the sex-specific median. For other components, a score of one was assigned to intake above or equal to the sex-specific median. Regarding alcohol, moderate intake (i.e., 10–50 g/d for men and 5–25 g/d for women) was assigned a score of one. A score of nine reflected maximum adherence. The IMD index evaluates the level of compliance to the MD, adapted to Italian eating behaviour. It consists of 11 components; high intakes of six typical Mediterranean foods (pasta, Mediterranean vegetables, fruit, legumes, olive oil, and fish), and low intakes of four non-Mediterranean foods (SSB, butter, red meat, and potatoes), and moderate consumption of alcohol (<12 g/day ethanol). Possible scores ranged from zero to 11 (28).

**Group C**

*Alternative Healthy Eating Index*

Three studies (19,29,30) in this review measured diet quality using the Alternative Healthy Eating Index 2010 (AHEI-2010), which is an alternative to the HEI-2005 and is based on foods and macronutrients predictive of chronic disease risk (31). It consists of 11 components; six of which focus on adequacy of the diet (vegetables, fruits, whole grains, nuts and legumes, long-chain omega-3 fatty acids, and poly-unsaturated fatty acids) and the remaining five focus on moderation (SSB and fruit juices, red and processed meat, trans fatty acids, sodium, and alcohol). The sum of components resulted in an overall score ranging from zero to a maximum achievable score of 110. One study used a modified version of and omitted the trans fatty acids component, as this information was not available (19).

*Dietary Approaches to Stop Hypertension Diet Score*

One study used the original Fung’s score which includes eight components; five on food groups to encourage (fruits, vegetables, nuts and legumes, whole grains, and low-fat dairy), and three of foods to be discouraged (red and processed meat, SSB and sodium). For each of the components, individuals are classified into sex-specific quintiles according to their intake. The overall score had a range of eight to 40, and higher scores indicate a diet which has greater accordance to the DASH pattern (32). The study using this score (10) only considered Na incorporated in food and not salt added during cooking or at the table for the sodium component. The other study used a modified DASH score (23), which considered UK dietary habits and included all non-milk extrinsic sugars in the diet, whereas the original score was limited to SSB (33). The score range remained unchanged.

*Comprehensive Diet Quality Index*

The comprehensive Diet Quality Index (cDQI) assesses the quality of both plant- and animal-sourced foods in the diet, and was reported by one study (34). The cDQI consists of 17 components; 11 plant-based foods (six healthful and five unhealthful) and six animal-sourced foods (three under each). Points are awarded based on intake of healthful plant-based foods (whole grains, vegetables (excluding white potatoes), whole fruits, nuts, seeds and legumes, vegetable oils and coffee and tea), or animal foods (fish and seafood, dairy products and poultry). Further, low intake of unhealthful plant-based (refined grains, fruit juices, SSB, and sweets and desserts) or animal foods (processed meats, unprocessed red meats, and eggs) are awarded points. It is composed of a plant-based DQI sub-score (pDQI) (ranging from 0 to 55) and an animal-based DQI sub-score (aDQI) (ranging from 0 to 30). The total cDQI total score, combining both pDQI and aDQI ranges from zero to 85. A higher pDQI, aDQI and cDQI score indicates a higher quality of plant-based foods, animal foods and both foods, respectively (35).

*Health Diet Indicator*

To quantify adherence to the 2002 WHO’s guidelines for prevention of chronic diseases, the Health Diet Indicator tool was developed. It consists of seven components based on the guidelines (saturated fatty acids, polyunsaturated fatty acids, cholesterol, protein, dietary fibre, fruits and vegetables and free sugars). A point is awarded when intake is within recommended WHO range, or if not, assigned zero, with the sum of all components ranging from zero (minimal adherence) to seven (maximal adherence) (36). Adherence to the guidelines was assessed in both the UK (23) and The Netherlands (10).

**Group D**

*Eat Lancet Diet*

Three studies measured adherence to the Eat Lancet Diet (ELD), one used the original score (37), which measures how close a diet is to the ELD reference recommendations (38). The score was constructed considering possible intake ranges based on recommendations. It consists of 14 components (whole grains, tubers and starchy vegetables, vegetables, fruits, dairy foods, beef, lamb, pork, chicken or other poultry, eggs, fish, legumes, soy foods, nuts, added fats, and added sugars). Individuals are assigned a point for meeting the cut-offs of each component, resulting in a possible range of zero to 14, with 14 representing perfect adherence (39). Two studies used the EAT-Lancet Diet Index (ELD-I) (21,40), which adapted the original score using the same cut-offs for each of the 14 components of the ELD. The ELD-I is based on continuous scoring of each component to improve discriminant power and account for deviation from the cut-off value, as opposed to discrete or binary scoring for each component (40). The calculation is energy-adjusted (2500kcal) and allocates positive points if consumption to promote is above the reference, and if the consumption of the food group to limit is below the reference. Conversely, it allocates negative points if the consumption of food groups to promote is below the reference, and if the consumption to limit is above the reference. The computing of the ELD-I leads to a continuous variable (positive or negative). The score of a diet that meets the reference is zero. One of the study used intakes of saturated and unsaturated fatty acids for the added fat component, as opposed to intake of saturated and unsaturated oils (21).

# **Table S2 AXIS Critical Appraisal of Cross-Sectional Studies**

| **Lead Author** | **Year** | **Introduction (1)** | **Methods (10)** | **Results (5)** | **Discussion (2)** | **Other (2)** | **Overall Quality** |
| --- | --- | --- | --- | --- | --- | --- | --- |
| Perraud | 2023 | 1 | 7 | 4 | 2 | 1 | 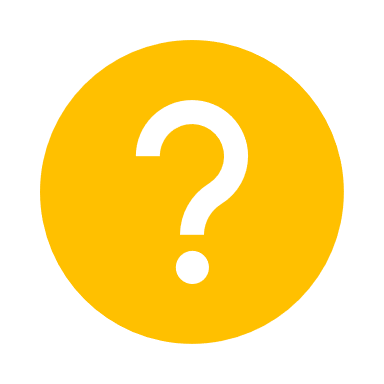 |
| Kesse-Guyot | 2022 | 1 | 7 | 4 | 2 | 2 | 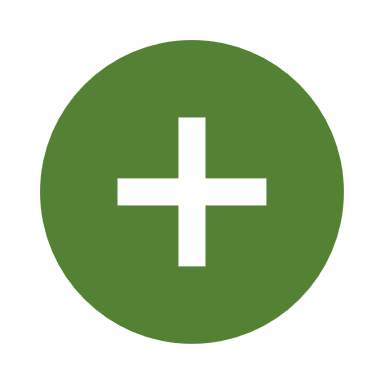 |
| Marty | 2022 | 1 | 5 | 4 | 2 | 2 | 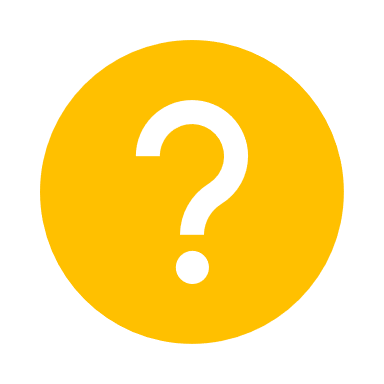 |
| Ridoutt | 2022 | 1 | 7 | 4 | 2 | 0 | 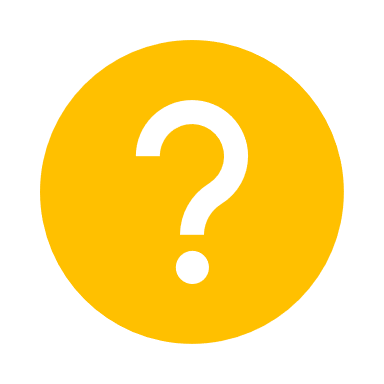 |
| Frehner | 2022 | 1 | 5 | 3 | 2 | 1 | 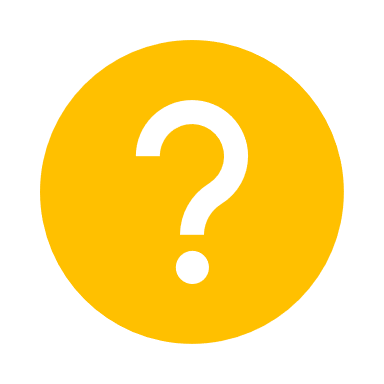 |
| Heerschop | 2021 | 1 | 7 | 4 | 2 | 1 | 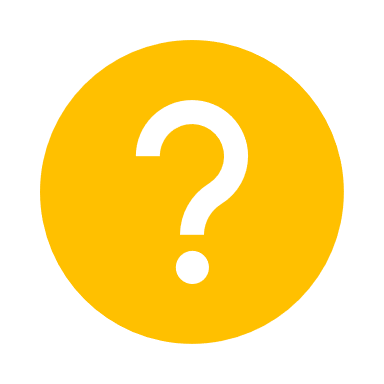 |
| Kesse-Guyot | 2021 | 1 | 7 | 3 | 2 | 2 | 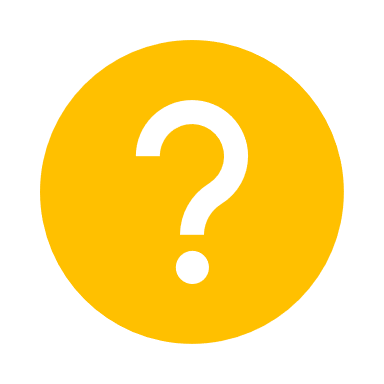 |
| Laine | 2021 | 1 | 7 | 3 | 2 | 2 | 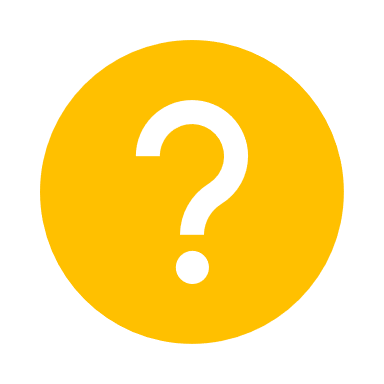 |
| Ridoutt | 2021 | 1 | 8 | 4 | 2 | 1 | 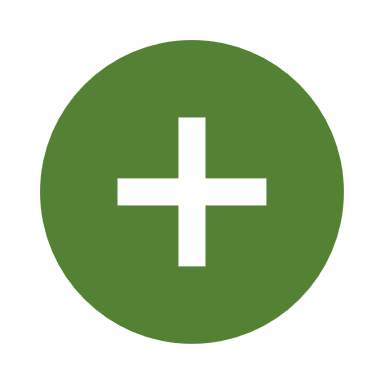 |
| Telleria-Aramburu | 2021 | 1 | 6 | 4 | 2 | 2 | 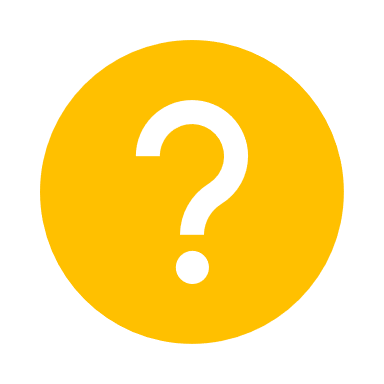 |
| Hobbs | 2021 | 1 | 8 | 4 | 2 | 1 | 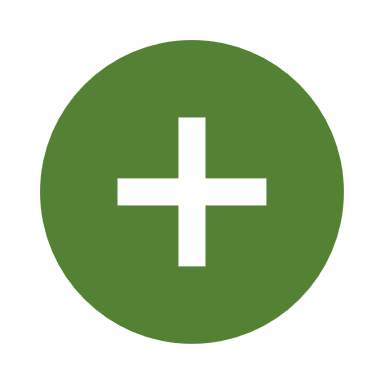 |
| Kesse-Guyot | 2020 | 1 | 7 | 4 | 2 | 2 | 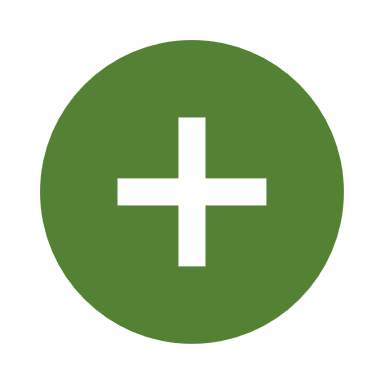 |
| van Bussel | 2020 | 1 | 6 | 3 | 2 | 2 | 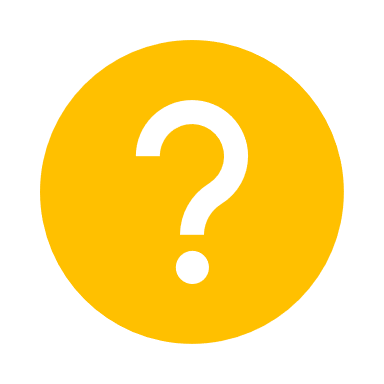 |
| Baudry | 2020 | 1 | 5 | 4 | 2 | 2 | 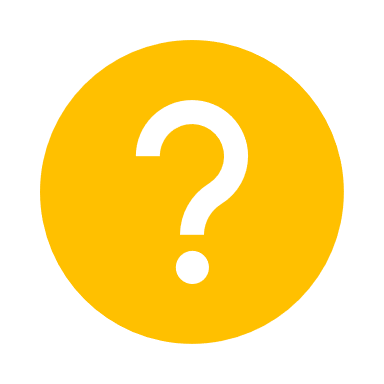 |
| Mertens | 2019 | 1 | 7 | 4 | 2 | 2 | 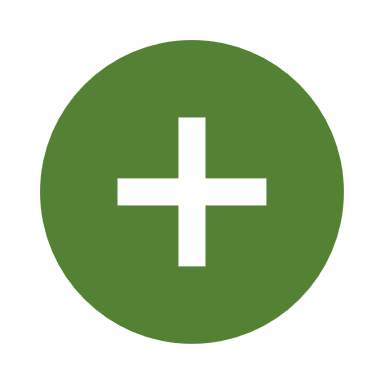 |

| van Bussel | 2019 | 1 | 8 | 4 | 2 | 1 | 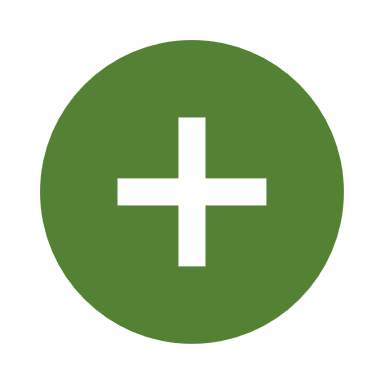 |
| --- | --- | --- | --- | --- | --- | --- | --- |
| Vellinga | 2019 | 1 | 8 | 3 | 2 | 1 | 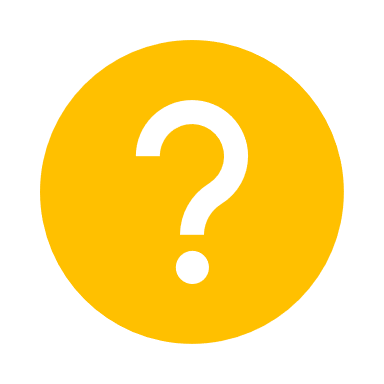 |
| Biesbroek | 2018 | 1 | 9 | 4 | 2 | 1 | 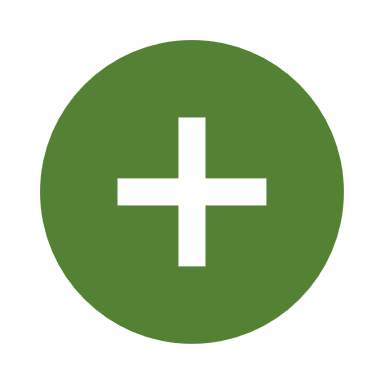 |
| Murakami | 2018 | 1 | 9 | 3 | 2 | 2 | 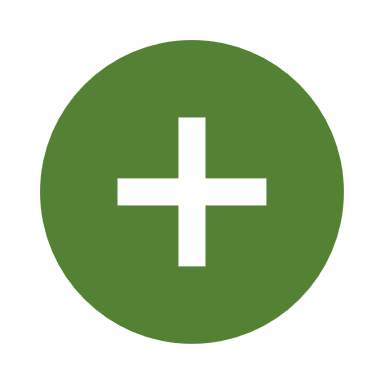 |
| Seconda | 2018 | 1 | 6 | 4 | 2 | 2 | 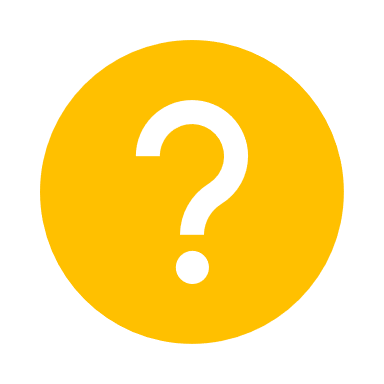 |
| Biesbroek | 2017 | 1 | 7 | 4 | 2 | 2 | 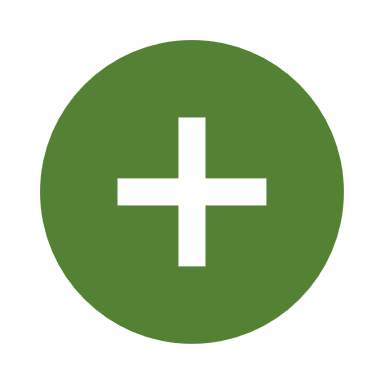 |
| Rosi | 2017 | 1 | 7 | 3 | 2 | 2 | 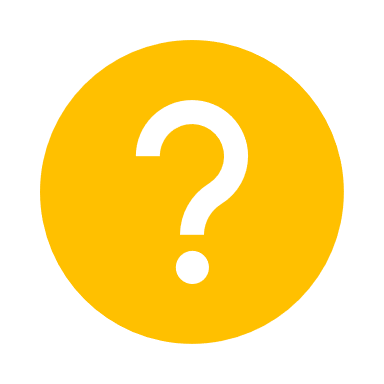 |

For each question, 1 point is awarded for a ‘yes’ response, whereas 0 points are awarded otherwise (i.e., a ‘no’ or ‘don’t know’ response). Each study was given a score ranging from 0 to 20. Two AXIS questions (Q19, Q20) were formulated such that a positive answer “yes” would reflect negatively on methodological quality. Therefore, the scoring of these two questions was reversed in order to provide a uniform scoring method. Based on the total scores, an overall subjective rating of quality was assigned; low quality (<10 points), fair quality (10-15 points), or good quality (≥16 points).

Denotes:
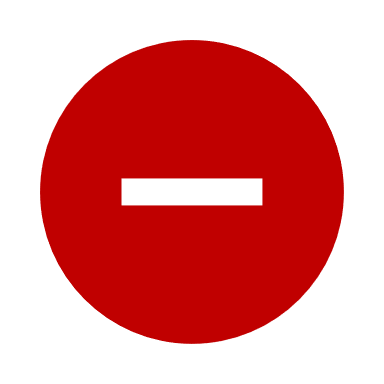
 Low quality,
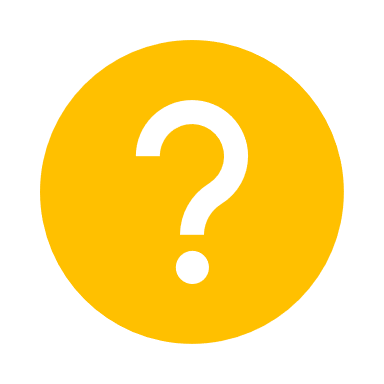
 Fair quality,
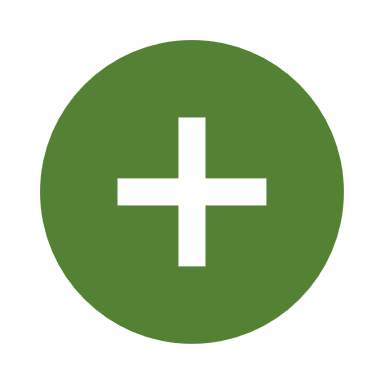
 Good quality

# **Table S3 NIH Quality Assessment Tool for Observational Cohort and Cross-sectional Studies**

| **Lead Author** | **Year** | **Introduction (1)** | **Methods (4)** | **Exposure (5)** | **Outcome (2)** | **Other (2)** | **Overall Quality** |
| --- | --- | --- | --- | --- | --- | --- | --- |
| Marty | 2022 | 1 | 2 | 2 | 1 | 1 | 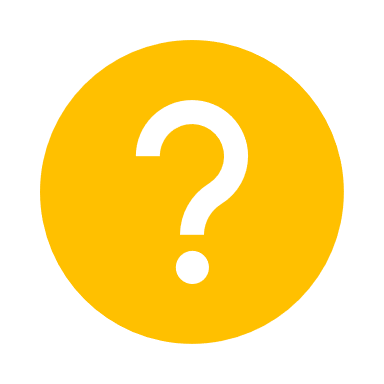 |
| Biesbroek | 2019 | 1 | 3 | 3 | 1 | 1 | 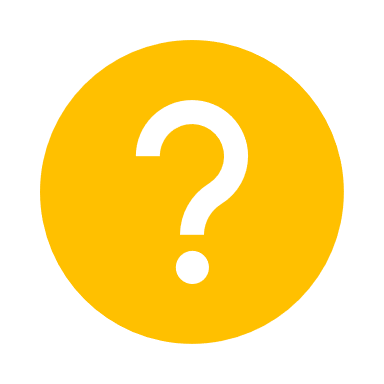 |

For each question, 1 point is awarded for a ‘yes’ response, whereas 0 points are awarded otherwise (i.e., a ‘no’ or ‘cannot determine’ or ‘not applicable’ or ‘not reported’ response). Each study was given a score ranging from 0 to 14. Based on the total scores, an overall subjective rating of quality was assigned; low quality (<5 points), fair quality (5–9 points) or good quality (≥10 points).

Denotes:
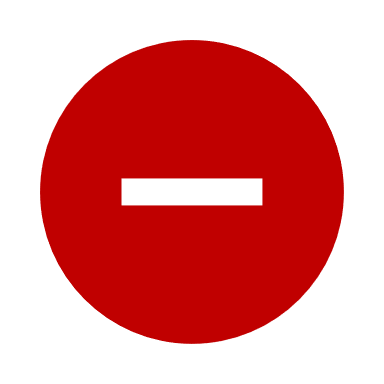
 Low quality,
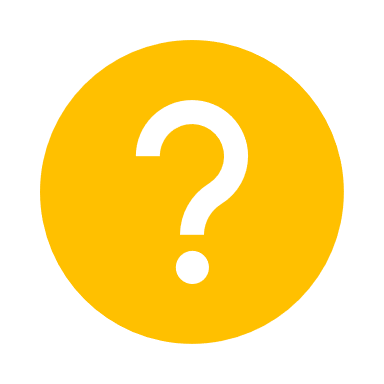
 Fair quality,
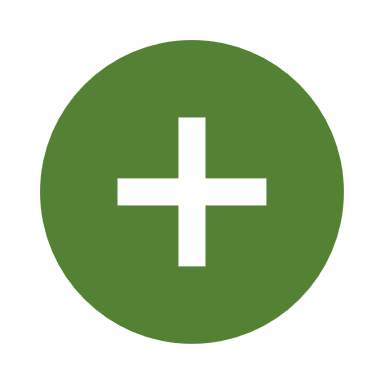
 Good quality

# **References**

1. Golley RK, Hendrie GA. Dietary Guideline Index for Children and Adolescents: What is the impact of the new dietary guidelines? Nutr Diet 2014;71:210–2.

2. Ridoutt B, Baird D, Hendrie GA. Diets with Higher Vegetable Intake and Lower Environmental Impact: Evidence from a Large Australian Population Health Survey. Nutrients 2022;14:1517.

3. Ridoutt BG, Baird D, Hendrie GA. The role of dairy foods in lower greenhouse gas emission and higher diet quality dietary patterns. Eur J Nutr 2021;60:275–85.

4. Looman M, Feskens EJ, Rijk M de, Meijboom S, Biesbroek S, Temme EH, Vries J de, Geelen A. Development and evaluation of the Dutch Healthy Diet index 2015. Public Health Nutr Cambridge University Press; 2017;20:2289–99.

5. Heerschop SN, Biesbroek S, Temme EHM, Ocké MC. Can Healthy and Sustainable Dietary Patterns That Fit within Current Dutch Food Habits Be Identified? Nutrients 2021;13:1176.

6. van Bussel LM, van Rossum CT, Temme EH, Boon PE, Ocké MC. Educational differences in healthy, environmentally sustainable and safe food consumption among adults in the Netherlands. Public Health Nutr 2020;23:2057–67.

7. van Bussel LM, Kuijsten A, Mars M, Feskens EJM, van ’t Veer P. Taste profiles of diets high and low in environmental sustainability and health. Food Qual Prefer 2019;78:103730.

8. Vellinga RE, van de Kamp M, Toxopeus IB, van Rossum CTM, de Valk E, Biesbroek S, Hollander A, Temme EHM. Greenhouse Gas Emissions and Blue Water Use of Dutch Diets and Its Association with Health. Sustainability 2019;11:6027.

9. Biesbroek S, Monique Verschuren WM, van der Schouw YT, Sluijs I, Boer JMA, Temme EHM. Identification of data-driven Dutch dietary patterns that benefit the environment and are healthy. Clim Change 2018;147:571–83.

10. Biesbroek S, Verschuren WMM, Boer JMA, Kamp ME van de, Schouw YT van der, Geelen A, Looman M, Temme EHM. Does a better adherence to dietary guidelines reduce mortality risk and environmental impact in the Dutch sub-cohort of the European Prospective Investigation into Cancer and Nutrition? Br J Nutr 2017;118:69–80.

11. Biesbroek S, Verschuren WM, Boer JM, Schouw YT van der, Sluijs I, Temme EH. Are our diets getting healthier and more sustainable? Insights from the European Prospective Investigation into Cancer and Nutrition – Netherlands (EPIC-NL) cohort. Public Health Nutr 2019;22:2931–40.

12. Telleria-Aramburu N, Bermúdez-Marín N, Rocandio AM, Telletxea S, Basabe N, Rebato E, Arroyo-Izaga M. Nutritional quality and carbon footprint of university students’ diets: results from the EHU12/24 study. Public Health Nutr 2022;25:183–95.

13. Guenther PM, Casavale KO, Reedy J, Kirkpatrick SI, Hiza HAB, Kuczynski KJ, Kahle LL, Krebs-Smith SM. Update of the Healthy Eating Index: HEI-2010. J Acad Nutr Diet 2013;113:569–80.

14. Estaquio C, Kesse-Guyot E, Deschamps V, Bertrais S, Dauchet L, Galan P, Hercberg S, Castetbon K. Adherence to the French Programme National Nutrition Santé Guideline Score Is Associated with Better Nutrient Intake and Nutritional Status. J Am Diet Assoc 2009;109:1031–41.

15. Kesse-Guyot E, Chaltiel D, Wang J, Pointereau P, Langevin B, Allès B, Rebouillat P, Lairon D, Vidal R, Mariotti F, et al. Sustainability analysis of French dietary guidelines using multiple criteria. Nat Sustain 2020;3:377–85.

16. Baudry J, Pointereau P, Seconda L, Vidal R, Taupier-Letage B, Langevin B, Allès B, Galan P, Hercberg S, Amiot M-J, et al. Improvement of diet sustainability with increased level of organic food in the diet: findings from the BioNutriNet cohort. Am J Clin Nutr 2019;109:1173–88.

17. Seconda L, Baudry J, Allès B, Boizot-Szantai C, Soler L-G, Galan P, Hercberg S, Langevin B, Lairon D, Pointereau P, et al. Comparing nutritional, economic, and environmental performances of diets according to their levels of greenhouse gas emissions. Clim Change 2018;148:155–72.

18. Kesse-Guyot E, Allès B, Brunin J, Fouillet H, Dussiot A, Mariotti F, Langevin B, Berthy F, Touvier M, Julia C, et al. Nutritionally adequate and environmentally respectful diets are possible for different diet groups: an optimized study from the NutriNet-Santé cohort. Am J Clin Nutr 2022;nqac253.

19. Perraud E, Wang J, Salomé M, Mariotti F, Kesse-Guyot E. Dietary protein consumption profiles show contrasting impacts on environmental and health indicators. Sci Total Environ 2023;856:159052.

20. Marty L, de Lauzon-Guillain B, Nicklaus S. Short- and Mid-Term Impacts of COVID-19 Outbreak on the Nutritional Quality and Environmental Impact of Diet. Front Nutr 2022;9:838351.

21. Marty L, Chambaron S, de Lauzon-Guillain B, Nicklaus S. The motivational roots of sustainable diets: Analysis of food choice motives associated to health, environmental and socio-cultural aspects of diet sustainability in a sample of French adults. Clean Responsible Consum 2022;5:100059.

22. Chaltiel D, Adjibade M, Deschamps V, Touvier M, Hercberg S, Julia C, Kesse-Guyot E. Programme National Nutrition Santé – guidelines score 2 (PNNS-GS2): development and validation of a diet quality score reflecting the 2017 French dietary guidelines. Br J Nutr 2019;122:331–42.

23. Murakami K, Livingstone MBE. Greenhouse gas emissions of self-selected diets in the UK and their association with diet quality: is energy under-reporting a problem? Nutr J 2018;17:27.

24. Rosi A, Mena P, Pellegrini N, Turroni S, Neviani E, Ferrocino I, Di Cagno R, Ruini L, Ciati R, Angelino D, et al. Environmental impact of omnivorous, ovo-lacto-vegetarian, and vegan diet. Sci Rep 2017;7:6105.

25. Sofi F, Macchi C, Abbate R, Gensini GF, Casini A. Mediterranean diet and health status: an updated meta-analysis and a proposal for a literature-based adherence score. Public Health Nutr 2014;17:2769–82.

26. Panagiotakos DB, Pitsavos C, Stefanadis C. Dietary patterns: A Mediterranean diet score and its relation to clinical and biological markers of cardiovascular disease risk. Nutr Metab Cardiovasc Dis 2006;16:559–68.

27. Trichopoulou A, Orfanos P, Norat T, Bueno-de-Mesquita B, Ocké MC, Peeters PH, Schouw YT van der, Boeing H, Hoffmann K, Boffetta P, et al. Modified Mediterranean diet and survival: EPIC-elderly prospective cohort study. Br Med J 2005;330:991.

28. Agnoli C, Krogh V, Grioni S, Sieri S, Palli D, Masala G, Sacerdote C, Vineis P, Tumino R, Frasca G, et al. A Priori–Defined Dietary Patterns Are Associated with Reduced Risk of Stroke in a Large Italian Cohort. J Nutr 2011;141:1552–8.

29. Frehner A, Zanten HHEV, Schader C, Boer IJMD, Pestoni G, Rohrmann S, Muller A. How food choices link sociodemographic and lifestyle factors with sustainability impacts. J Clean Prod 2021;300:126896.

30. Hobbs DA, Durrant C, Elliott J, Givens DI, Lovegrove JA. Diets containing the highest levels of dairy products are associated with greater eutrophication potential but higher nutrient intakes and lower financial cost in the United Kingdom. Eur J Nutr 2020;59:895–908.

31. Chiuve SE, Fung TT, Rimm EB, Hu FB, McCullough ML, Wang M, Stampfer MJ, Willett WC. Alternative Dietary Indices Both Strongly Predict Risk of Chronic Disease. J Nutr 2012;142:1009–18.

32. Fung TT, Chiuve SE, McCullough ML, Rexrode KM, Logroscino G, Hu FB. Adherence to a DASH-style diet and risk of coronary heart disease and stroke in women. Arch Intern Med United States; 2008;168:713–20.

33. Penney TL, Jones NRV, Adams J, Maguire ER, Burgoine T, Monsivais P. Utilization of Away-From-Home Food Establishments, Dietary Approaches to Stop Hypertension Dietary Pattern, and Obesity. Am J Prev Med 2017;53:e155–63.

34. Kesse-Guyot E, Allès B, Brunin J, Fouillet H, Dussiot A, Mariotti F, Langevin B, Berthy F, Touvier M, Julia C, et al. Nutritionally adequate and environmentally respectful diets are possible for different diet groups: an optimized study from the NutriNet-Santé cohort. Am J Clin Nutr 2022;116:1621–33.

35. Keaver L, Ruan M, Chen F, Du M, Ding C, Wang J, Shan Z, Liu J, Zhang FF. Plant- and animal-based diet quality and mortality among US adults: a cohort study. Br J Nutr 2021;125:1405–15.

36. World Health Organization. Diet, nutrition, and the prevention of chronic diseases: report of a joint WHO/FAO expert consultation [Internet]. Geneva, Switzerland: WHO; 2003. Available from: https://apps.who.int/iris/handle/10665/42665

37. Laine JE, Huybrechts I, Gunter MJ, Ferrari P, Weiderpass E, Tsilidis K, Aune D, Schulze MB, Bergmann M, Temme EH. Co-benefits from sustainable dietary shifts for population and environmental health: an assessment from a large European cohort study. Lancet Planet Health 2021;5:e786–96.

38. Willett W, Rockström J, Loken B, Springmann M, Lang T, Vermeulen S, Garnett T, Tilman D, DeClerck F, Wood A, et al. Food in the Anthropocene: the EAT–Lancet Commission on healthy diets from sustainable food systems. The Lancet 2019;393:447–92.

39. Knuppel A, Papier K, Key TJ, Travis RC. EAT-Lancet score and major health outcomes: the EPIC-Oxford study. Lancet 2019;394:213–4.

40. Kesse-Guyot E, Rebouillat P, Brunin J, Langevin B, Allès B, Touvier M, Hercberg S, Fouillet H, Huneau J-F, Mariotti F, et al. Environmental and nutritional analysis of the EAT-Lancet diet at the individual level: insights from the NutriNet-Santé study. J Clean Prod 2021;296:126555.
